# Targeting KRAS mutations: orchestrating cancer evolution and therapeutic challenges

**DOI:** 10.1038/s41392-025-02473-8

**Published:** 2025-11-28

**Authors:** Khalil Choucair, Hafsa Imtiaz, Md Hafiz Uddin, Misako Nagasaka, Mohammad Najeeb Al-Hallak, Philip A. Philip, Bassel El-Rayes, Boris C. Pasche, Asfar S. Azmi

**Affiliations:** 1 Department of Oncology, Wayne State University School of Medicine, Detroit, MI USA; 2 Department of Division of Hematology/Oncology and Medicine, School of Medicine University of California Irvine, Irvine, CA USA; 3 Henry Ford Health Systems, Detroit, MI USA; 4 Department of Internal Medicine, Department of Hematology and Oncology, University of Alabama at Birmingham, Birmingham, AL USA

**Keywords:** Drug development, Oncogenes

## Abstract

Activating *KRAS* mutations are highly relevant to various cancers, and KRAS is the most frequently altered oncogenic protein in solid tumors. While historically considered undruggable, two KRAS^G12C^ inactive state-selective inhibitors are currently approved for treating patients with non-small cell lung cancer. However, these agents only demonstrate a 30–40% response rate and a median progression-free survival of approximately 6 months, with the inevitable emergence of resistance mechanisms, hence remaining far from achieving a cure. Additionally, several cancers with poor prognostic outcomes, such as pancreatic adenocarcinoma, are driven by other non-G12C *KRAS* mutations and thus have no effective targeted therapies. Improvements in understanding RAS signaling, RNA, and nucleic acid chemistry, as well as the role of the tumor microenvironment, have sparked a paradigm shift in the approach to KRAS inhibition and suggested the potential for several novel combination therapies. In this review, we provide an overview of the RAS pathway and discuss the ongoing development and status of therapeutic strategies for targeting the oncogenic RAS. We further delve into the challenges of resistance mechanisms to better understand the rationale behind these developing strategies, describe their mechanisms of action, and offer insights into the current clinical trial status of each of these approaches.

## Introduction

Kirsten rat sarcoma viral oncogene homolog (*KRAS*) is an isoform of the *Ras* superfamily of proteins, which also includes Harvey rat sarcoma virus oncogene (*HRAS*) and neuroblastoma RAS virus oncogene homolog (*NRAS*). KRAS proteins are known for their intrinsic GTPase activity and are “the most mutated protein in cancer”.^1^ *KRAS* mutation is foundational to in vivo oncogenic transformation^2,3^ and is widely associated with pancreatic ductal adenocarcinoma (PDAC), colorectal cancer (CRC), non-small cell lung cancer (NSCLC), cholangiocarcinoma, and uterine endometrial carcinoma.^1–3^ Approximately 30% of all human cancers harbor *RAS* mutations, with *KRAS* mutations being prevalent. *RAS* genes have tissue-specific profiles, which explains the predilection of KRAS for PDAC, CRC, and NSCLC. *NRAS* mutations are observed specifically in cutaneous melanoma, whereas *HRAS mutations* are detected in head and neck squamous cell carcinoma.^3^

**Fig. 1:**
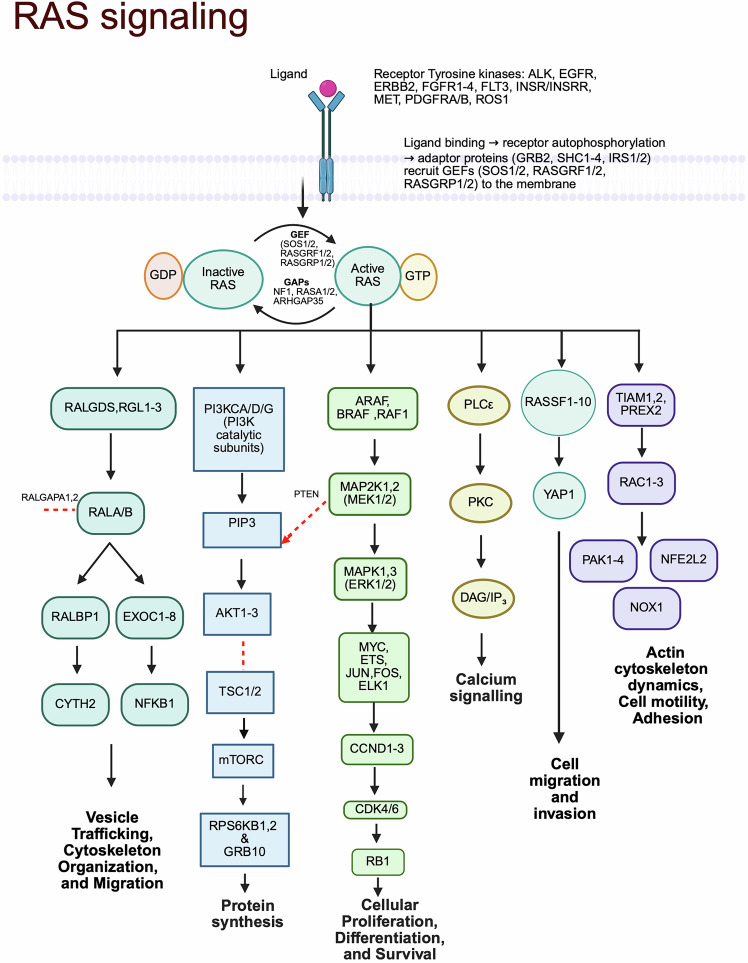
The KRAS signaling pathway and downstream effectors. The binding of epidermal (EGF) and platelet-derived (PDGF) growth factors to transmembrane receptors activates the intracellular phosphorylation (P) of inactive guanine diphosphate (GDP)-bound KRAS to its active triphosphate (GTP) form via guanine-exchange factor (GEF). Active wild-type KRAS subsequently activates downstream signaling pathways, including the RAS/MEK/ERK and PI3K/AKT/mTOR pathways, among others, thus leading to increased survival, proliferation, migration, differentiation, and invasion. Created in BioRender.com

All RAS isoforms are membrane-bound, small monomeric G proteins, with a highly conserved catalytic region responsible for nucleotide exchange.^4,5^ KRAS proteins operate as GDP-GTP-regulated on-off switches,^3^ which are regulated by guanine nucleotide exchange factors (GEFs), such as SOS, and GTPase-activating proteins (GAPs), such as neurofibromin 1 (NF1). When cells are activated by relevant stimuli, GEFs promote a KRAS-GTP (on) configuration, culminating in the activation of downstream RAS effector pathways, including RAF-MEK-ERK, PI3K-AKT-mTOR, and RALGD pathways.^6^ Oncogenic transformation of the *KRAS* gene allows increased signaling via the MAPK and PI3K/AKT pathways, culminating in increased cellular survival and proliferation. It also leads to the dysregulation of pathways that are responsible for cellular functions such as apoptosis, membrane vesicle trafficking, growth arrest and differentiation, cytoskeletal organization, calcium transport, and cell-cell junction signaling.^7^ Figure 1 summarizes the KRAS signaling pathway and its downstream effectors.

Despite its critical role in tumorigenesis, KRAS has long been considered an undruggable target for focused pharmacological therapy because of its inviolable structure. However, the identification of a new allosteric target in the switch pocket II region of the *KRAS G12C* mutation that can covalently bind with specific inhibitors and subsequently induce apoptosis paves the way for therapeutic developments of the first *KRAS* inhibitors.^8,9^ The FDA has since approved AMG510 (sotorasib) and MTRX849 (adagrasib) for the treatment of lung adenocarcinoma.^10^ These developments have led to increased vigor in the development of inhibitors specifically targeting KRAS. In this review, we provide a brief overview of KRAS mutations in cancers and tumorigenesis, specifically focusing on currently approved therapies for targeting KRAS, ongoing advances, resistance mechanisms, and novel combinational approaches.

## *KRAS* mutations in cancer: summary of research history and milestones

**Fig. 2:**
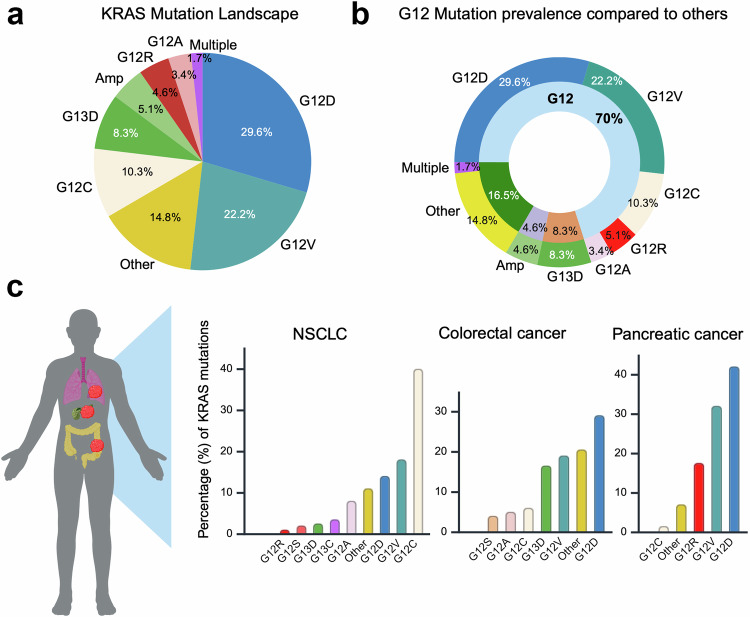
Prevalence of KRAS mutations. **a** Describes the prevalence (%) of the different types of KRAS mutations and alterations across all tumor types. **b** Further describes the frequency (%) of G12 mutations in comparison to other non-G12 KRAS mutations and alterations. **c** depicts the prevalence (y-axis-%) of specific KRAS mutations (x-axis) in non-small cell lung cancer (NSCLC), colorectal cancer, and pancreatic cancer. Created in BioRender.com

**Fig. 3:**
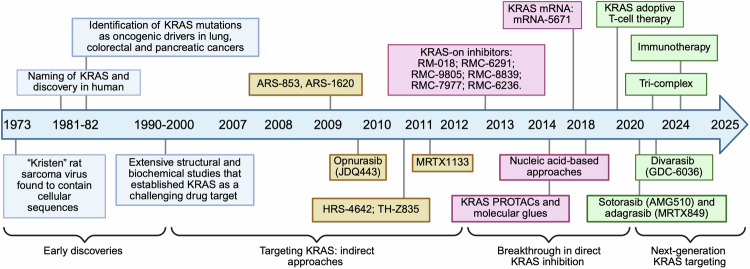
Historical timeline of major milestones in KRAS discovery and therapeutic development (1973–2025). This timeline charts the evolution of KRAS research from its identification in the Kristen rat sarcoma virus to the advent of next-generation therapies. Early decades defined KRAS biology and its role as a crucial oncogenic driver. The 2010 era marked a paradigm shift with the emergence of direct KRASG12C inhibitors, followed by diversification of targeting strategies into nucleic acid therapeutics, PROTACs, and immunotherapies. Current efforts focus on multimodal and next-generation strategies aimed at overcoming resistance and expanding therapeutic reach. Created in BioRender.com

*KRAS* is among the most frequently mutated oncogenes in human cancers, particularly in lung, colorectal, and pancreatic cancers.^11^ Most mutations are gain-of-function and single-base missense changes, with 98% occurring at codons 12 (G12), 13 (G13), and 61 (Q61).^2^ Codon G12 mutations are the most common, producing 3 distinct mutant subtypes: G12D (29.19%), G12V (22.17%), and G12C (13.43%).^6^ According to the Cancer Genome Atlas (TCGA) data, *KRAS* mutations appear in approximately 11.2% of all cancers, with higher rates (up to 45%) reported in other studies depending on cancer type. They are most prevalent in patients with PDAC (82.1%), with G12D accounting for 37.0% of those patients. CRC follows (~40%), with the G12D (12.5%) and G12V (8.5%) mutant variants being the most prevalent. In NSCLC, 21.20% of patients harbor *KRAS* mutations, primarily G12C (13.6%). Lower frequencies are observed in cholangiocarcinoma (12.7%), uterine endometrial carcinoma (14.1%), testicular germ cell tumor (11.7%), and cervical squamous cell carcinoma (4.3%). Figure 2 summarizes the prevalence of *KRAS* mutations across solid malignancies. Owing to their strong oncogenic role, especially in lethal cancers such as PDAC, NSCLC, and CRC, *KRAS* mutations have been intensively studied over the past 40 years. This section reviews key research milestones, leading to the most recent approvals of KRAS inhibitors and their clinical indications. Figure 3 provides a timeline summary of these research endeavors.

### KRAS structural and functional characterization: a historical perspective

Our understanding of the role of KRAS in tumorigenesis began in 1982 when point mutations in human RAS orthologs were identified as oncogenic drivers in human cancer cell lines.^12–20^ Cooper et al. first reported a transforming human DNA fragment, homologous to viral KRAS, in LX-1, a lung cancer cell line.^12^ Similar findings were independently reported in additional human lung, colon, gallbladder, pancreas, and rhabdomyosarcoma cell lines.^21^ Sequencing of vial *KRAS* and cloning of the transforming fragments led to the identification of an activating point mutation in codon 12 in both lung (Calu-1) and colorectal (SW480) cancer cell lines.^22^ These mutations introduce amino acid substitutions, confirming that KRAS is a proto-oncogene.^18–20^

Research has subsequently focused on the functional structure and biochemical activity of KRAS to enable drug targeting. However, extensive structural and biochemical studies have established that KRAS is a challenging drug target (reviewed in ref. ^23^). In the late 1980s, studies showed that epidermal growth factor (EGF) binding to its respective receptor (EGFR) stimulated guanine nucleotide binding to RAS but that oncogenic RAS activation was EGFR independent.^24,25^ Subsequent work further characterized the involvement of RAS proteins in the activation of phospho-inositol phosphate (PIP) signaling pathways.^26,27^

The discovery of GEFs, initially in yeast models, was key to understanding RAS signaling biology and activity.^28–30^ The subsequent isolation of SOS in Drosophila and mammalian models established the regulatory role of GEFs as cellular activators of RAS GTPases via the modulation of nucleotide binding to RAS proteins.^31,32^ Another important discovery that shaped our understanding of RAS regulation was described in 1987 by Trahe and McCormick: using mouse and human cell lines, a protein capable of inactive RAS GTPase activity was identified and shown to act through inducing a factorial increase in the intrinsic GTP hydrolytic activity of cellular RAS proteins.^33,34^ This protein was named GTPase-activating protein (GAP). Interestingly, oncogenic mutations in RAS render the RAS protein insensitive to GAP. Since this initial discovery, 14 other mammalian RAS GAPs have been discovered, including NF1, GAP1, and SynGAP.^35,36^ The molecular dissection and elucidation of RAS signaling further characterized GRB2, an adapter protein that acts as an intermediate link between surface receptors and RAS activation, through the intracellular binding of phosphorylated tyrosine residues on transmembrane receptors on the one hand and RAS/SOS on the other hand, hence triggering GEP exchange activity on RAS proteins at the plasma membrane.^37–40^ These discoveries led to the first description of the complete RAS pathway in 1994, starting with EF-dependent receptor activation, dimerization, and autophosphorylation; the recruitment of GRB2 and its associated SOS GEP proteins to the plasma membrane; the subsequent activation of RAS; and downstream intracellular signaling activation to the nucleus.^41^ The first downstream effector of RAS, RAF-1, was first described in 1993 and was shown to bind RAS only in its active GTP-bound state, leading to subsequent activation of the MAPK cascade.^42,43^ Other effectors, such as PI3K, PKCζ, and RALGDS, were subsequently described (reviewed in ref. ^44^), along with their downstream signaling, ultimately resulting in the regulation of protein synthesis, glucose transport, and proliferation.^45–49^

While the function of KRAS was understood, its structural characterization did not fully mature until 1998, when it was characterized through crystallographic studies.^50^ In early mutagenesis studies, functional domains were classified as “essential” if mutations within that region reduced the biological activity of RAS proteins and “dispensable” if not.^51^ Through such mutagenic approaches, specific regions responsible for RAS-GAP interactions and for binding guanine nucleotides have been identified.^50,52^ The first crystal structure published in 1988 included four α-helixes and six β-sheets connected by nine loops and confirmed the spatial locations of the functional domains previously identified by mutagenesis. Later studies precisely defined the amino acids responsible for GTP binding, as well as the relevance of the switch-I and switch-II pockets in RAS-GEF interactions.^50,53–59^

### KRAS as a therapeutic target: clinical research endeavors toward the approval of clinical indications

Early efforts to inhibit KRAS focused on blocking its membrane localization, which is essential for its activation.^60,61^ Farnesyltransferase inhibitors (FTIs) aim to prevent the lipid modification of the CAAX motif necessary for this localization.^62^ However, despite promising preclinical results, FTIs have shown limited clinical efficacy in *KRAS*-mutant cancers.^63–66^ Other strategies, including disrupting KRAS palmitoylation via calcium channel blockers; targeting proteins required for membrane localization, such as refs. ^67–69^; and targeting proteins required for KRAS membrane localization, such as PDEδ, have also failed in clinical trials (NCT00005989 and NCT00005648)^70,71^ (reviewed in ref. ^72^).

Owing to these limitations, attention has shifted to indirect targeting of KRAS through the inhibition of its downstream effectors, particularly the RAF-MEK-ERK-MAPK and PI3K/AKT/mTOR pathways. While effective in preclinical models, their clinical outcomes are often disappointing due to a combination of factors, including toxicity, compensatory mechanisms, and paradoxical signaling activation.^73,74^ For example, MAPK pathway inhibitors in KRAS-driven cancers have shown limited efficacy, along with significant toxicity and unexpected dynamic feedback mechanisms.^75,76^ Similarly, inhibition of the PI3K pathway via PI3K inhibitors (BKM120, GDC0941, XL147) in *KRAS*-mutated NSCLC resulted in modest efficacy, with preliminary data suggesting insensitivity to PI3K inhibition as a monotherapy, likely due to the activation of multiple alternative KRAS-activated pathways and their crosstalk.^77,78^

Because of the limitations of targeting downstream effectors, further research efforts have shifted toward direct targeting of KRAS itself. The discovery of a cryptic pocket (switch-II pocket) in KRAS G12C by Shokat and colleagues in 2013 was a major milestone in direct KRAS targeting, which was previously considered undruggable.^9^ Preclinical work has characterized the difference between G12C, on the one hand, and G12D and G12V *KRAS* mutations, on the other hand, in terms of their ability to maintain alternative interactions with their downstream effectors, although active cycling between the GDP-bound and GTP-bound states. This difference allowed early attempts to target and lock KRAS G12C in its inactive conformation, with the goal of overcoming the challenges of drug permeability and specificity.^79,80^ The subsequent identification of the thiol group in the cysteine residue that forms a disulfide bridge allowed the development of specific inhibitors with prolonged target engagement, remarkable selectivity, and greater activity, given the location of the cysteine 12 residue near the switch regions involved in the interaction with effectors and the nucleotide.^9^ The crystal structure of the initial inhibitor compound combined with the KRAS G12C-GDP complex revealed that the compound extended from the cysteine residue into an adjacent pocket, termed the switch-II pocket (S-IIP), rather than binding to the nucleotide pocket previously targeted by earlier compounds.^9^ This new targeting strategy had not been described before, and Shokat and colleagues pursued further structural modifications, including the introduction of acrylamides, vinyl sulfonamides, and carbon-based electrophiles, rather than continuing with disulfide-based compounds, thus increasing selectivity and, for the first time, irreversible inhibitory effects. The most potent acrylamide compound was tested in cells harboring the *KRAS* G12C mutation, which resulted in reduced viability and increased apoptosis.^9^

The discovery of this new allosteric pocket in KRAS G12C and relatively specific inhibitors provided the first structure-based validation that KRAS is targetable.^81^ On the basis of prior compounds described by Shokat and colleagues, renewed interest and efforts have emerged to develop novel compounds with potential for clinical success: ARS-853, for example, was developed by optimizing electrophilic localization and modifying the scaffold that interacts with a hydrophobic portion of S-IIP.^82,83^ The compound was shown to inhibit the KRAS G12C protein by locking it in an inactive state.^84,85^ ARS-1620 was the first proof-of-concept molecule tested in vivo and differed from ARS-853 in that it has an additional covalent interaction with the His95 residue of KRAS G12C, providing a more stable and preferred conformation.^86^ This new structure provides the basis for exploring AMG510 (sotorasib) in the clinical arena.

AMG510 is the first small-molecule inhibitor that specifically targets KRAS G12C to enter clinical trials (NCT03600883). The molecule specifically and irreversibly binds to the cysteine 12 residue in the inducible S-IIP pocket and locks KRAS G12C in its inactive state.^86,87^ Compared with ARS-1620, AMG510 has an additional novel surface groove via an alternative orientation of His95 on KRAS, thus resulting in a 10-fold improvement in efficacy.^88,89^ AMG510 received its first approval in 2021 as the first treatment for adult patients with *KRAS* G12C-mutated NSCLC who had received at least one prior line of systemic therapy.^90^ This milestone approval of the first inhibitor to directly target mutated KRAS was the culmination of several years of clinical evaluation: in the phase I trial of AMG510 monotherapy (NCT03600883), 129 patients received the drug and had responses across all dose levels, with no dose-limiting toxicity or treatment-related deaths.^91^ In terms of response, patients with NSCLC (n = 59) had an objective response rate (ORR) of 32.2%, a disease control rate (DCR) of 88.1%, and a median progression-free survival (PFS) of 6.3 months. The efficacy data and the recommended phase II dose (960 mg daily) supported the phase II trial of AMG510 (NCT03600883), which evaluated 126 patients with KRAS G12C-mutated NSCLC. In this trial, an ORR of 37.1%, including 3 complete responses and 43 partial responses, was reported, along with a DCR of 80.6% and a median PFS of 6.8 months, which is in line with the phase 1 results. The details of these trials, as well as other trials using AMG510, will be discussed later in the “Therapeutic strategies to target KRAS” section of this review. MRTX849 (adagrasib) is another KRAS inhibitor that successfully targets the KRAS G12C protein.^92^ This drug, with a slightly different chemical structure, also irreversibly and selectively binds and locks KRAS G12C in its inactive GDP-bound state, with a longer half-life than AMG510.^93^ MRTX849 is the second (and last) KRAS G12C direct inhibitor to be approved and is indicated as a 2nd-line treatment for metastatic NSCLC with the *KRAS G12C* mutation.^94^ This approval was based on the Krystal-1 (NCT03785249) trial, which we discuss in detail later in this review. Currently, AMG510 (sotorasib) and MTRX849 (adagrasib) are the only two approved KRAS G12C inhibitors. The next sections explore the central role of KRAS signaling, its regulatory mechanisms, the challenges of resistance, and, finally, therapeutic strategies.

## KRAS signaling pathways and molecular crosstalk

KRAS signaling is central to several pathways regulating cell growth, survival, and differentiation. Upon activation by upstream RTKs, KRAS triggers and regulates several downstream signaling pathways, which we review below.^73,84^

*In the RAF-MEK-ERK pathway*, activated KRAS directly activates the mitogen-activated protein kinase (MAPK) pathway by first recruiting and activating RAF kinases (RAF-1, BRAF, ARAF).^95^ Activated RAF, in turn, phosphorylates and activates MEK1/2, leading to downstream phosphorylation and activation of ERK1/2. ERK translocates to the nucleus and promotes transcriptional activation, resulting in increased proliferation, differentiation, and survival.^96^ On the one hand, ERK is also involved in molecular crosstalk through the inhibition of components of the PI3K pathway, and on the other hand, via negative feedback loops that suppress upstream RAF and RTKs.^97^

*In the PI3K-AKT-mTOR pathway*, activated KRAS interacts with phosphoinositide 3-kinase (PI3K), leading to the phosphorylation of phosphatidylinositol 4,5-bisphosphate (PIP2) into its active PIP3 form. PIP3 recruits and activates AKT, which phosphorylates (and inhibits) proapoptotic proteins (BAD, FOXO) while activating mTOR, a key regulator of protein synthesis and cell growth.^98^ The net effect is the upregulation of cell survival and metabolism. As described earlier, PI3K-AKT signaling can be modulated by the MAPK pathway through ERK-mediated inhibition of the PI3K pathway, and vice versa.^98,99^

*In the Ral-GEF pathway*, KRAS also activates Ral GEFs, leading to the activation of RalA and RalB.^100^ Ral proteins regulate cytoskeletal dynamics, resulting in increased vesicle trafficking and cell migration, which are necessary for metastasis.^101^ The Ral pathway modulates endocytosis and exocytosis processes that are also influenced by other KRAS-driven pathways (such as the PI3K‒AKT pathway).^102^

*The RAC1-PAK pathway*: RAS indirectly activates RAC1, a GTPase involved in cytoskeletal remodeling.^101^ Activated RAC1 results in the activation of PAK kinases involved in cell motility and survival.^103^ The RAC1/PAK signaling pathway, in turn, modulates both the MAPK and PI3K pathways, hence impacting cellular proliferation and metastasis.^104,105^

*The YAP/TAZ Hippo pathway*: Ras activation indirectly activates the YAP/TAZ Hippo pathway. It does so by inhibiting LATS kinases, thus leading to increased YAP/TAZ activity.^106^ LATS kinases are key components of the Hippo pathway and normally phosphorylate and inhibit YAP/TAZ. By inhibiting LATS kinases, RAS relieves YAP/TAZ from inhibition, leading to its nuclear translocation, resulting in the upregulation of genes involved in stemness, invasion, and drug resistance.^106,107^ RAS also interacts with Rho and RASSF, resulting in another indirect mechanism of YAP/TAZ activation. YAP/TAZ is also involved in molecular crosstalk and enhances MAP and PI3K signaling, hence reinforcing oncogenic effects.^108^

Given the central role of KRAS in regulating key regulatory cellular pathways, KRAS mutations that cause constitutive activation of KRAS result in uncontrolled downstream signaling. Continuous activation of the MAPK pathway and the resulting ERK activation promote unchecked proliferation and survival, often rendering tumors resistant to MAPK inhibitors due to feedback reactivation. Similarly, hyperactivation of the PI3K-AKT pathway contributes to metabolic reprogramming, increased survival, and therapeutic resistance, highlighting the potential value of dual targeting of MAPK and PI3K in *KRAS*-mutated cancer. Uncontrolled upregulation of the Ral-GEF and RAC1 pathways leads to enhanced invasion, cell motility, and metastasis. Finally, increased YAP/TAZ activation has been shown to enhance stem-like properties and therapy resistance in KRAS-driven tumors. Taken together, these observations highlight not only the central role of KRAS but also the plethora of potential therapeutic interventions that have been developed. Understanding the regulatory mechanisms of KRAS is key to developing such therapeutic strategies. In the next section, we review these regulatory mechanisms and their variation across tumor types before discussing currently approved and ongoing investigations of KRAS-targeted therapeutic strategies.

## KRAS regulatory mechanisms and impact of mutations on tumorigenesis

As a master regulator of multiple cancer-promoting pathways, KRAS itself is under tight regulatory control at different levels of the signaling pathway. In addition, as discussed earlier, KRAS controls major signaling pathways involved in tumorigenesis, highlighting the need to understand the clinical impact of mutated *KRAS* on cancer development and progression, as well as potential differences in prevalence and KRAS-related molecular heterogeneity across tumor types.

### KRAS regulatory mechanisms

The regulation of KRAS occurs mainly through molecular switch controls that alter the cycling between its activated and inactivated conformations.

Three main mechanisms/pathways upstream of KRAS are involved in KRAS regulation: (i) the GRB2-SOS1 complex, (ii) RAS-GRF1, and (iii) Src homology phosphatase 2 (SHP2).^109–119^ The GRB2-SOS1 complex is an intermediary that relays signals between RTKs and KRAS: upon activation of EGFR, growth factor receptor-bound protein 2 (GRB2) acts as an adapter molecule that binds phosphorylated EGFR via its SH2 domain and SOS1 through its SH3 domain.^37–39,109^ SOS1 itself is a GEF that is activated once bound to GRB2 and promotes the binding of GTP and KRAS, hence converting KRAS from an inactive state to an active state. More recently, novel mechanisms of KRAS activation independent of membrane activation have been described, whereby fusion proteins that contain portions of RTK can combine with GRB2-SOS to form a cytoplasmic protein granule that directly activates KRAS.^50,110^

RAS protein-specific guanine nucleotide releasing factor 1 (RAS-GRF1) is another key upstream regulator of KRAS that is expressed primarily in the brain.^111^ As a GEF, it connects molecular signals from glutamate receptors in mature neurons to KRAS, thereby promoting its activation and downstream MAPK/ERK cascade signaling.^111^ RAS-GRF1-mediated activation of KRAS is dependent on the calcium concentration, revealing mutual communication between KRAS and calcium signaling, as well as metabolism.^112,113^ RAS-GRF1 can also be activated by protein kinase A, itself under the regulation of the chemokine receptor-mediated production of cyclic AMP.^114^ Finally, SHP2 is a key molecule in KRAS activation.^115,116,120^ SHP2 is a protein tyrosine phosphatase (PTP) that uniquely acts as a common signaling hub between multiple signaling pathways and the KRAS-ERK pathway^117^: SHP2 acts as a scaffolding protein for GRB2, facilitating its recruitment, while also exerting the catalytic activity needed to promote the activation of KRAS through dephosphorylation of the SHP2 substrate.^118,119^ Some dephosphorylated substrates of SHP2 promote KRAS activation. SHP2 also indirectly activates KRAS by dephosphorylating p120-RASGAP, thus relieving its negative effect on KRAS.^121,122^

We have previously described downstream effector pathways that are directly controlled by KRAS activation, including the RAF-MEK-ERK, the PI3K-AKT-mTOR, and other signaling pathways. While they act mainly as effector pathways, they also exert feedback regulation on KRAS.

### KRAS mutations across tumors: differences in prevalence, biochemical heterogeneity, and impact of comutations

Despite the well-described structure, signaling, and regulation of KRAS in cancer, *KRAS* mutations are neither equally prevalent in different tumor types nor exert similar biochemical effects.

As described earlier, *KRAS* is one of the most frequently mutated oncogenes in human cancers, particularly in lung, colorectal, and pancreatic cancers.^11^ *KRAS* mutations are most prevalent in patients with PDAC, followed by patients with CRC or NSCLC. *KRAS* mutations are also observed in cholangiocarcinoma, uterine endometrial carcinoma, testicular germ cell tumors, and cervical squamous cell carcinoma.^74^ The three mutation subtypes (G12C, G12D, and G12V) are also differentially prevalent across tumor types (Fig. 2).

Pancreatic adenocarcinoma (PDAC): *KRAS* mutations are highly prevalent in PDAC, with some studies reporting that up to 90% of cases harbor mutations, with the G12D subtype being the most commonly reported.^123^ PDAC is characterized by high mortality rates and is the third leading cause of cancer-related deaths in the United States.^11^ *KRAS* oncogene activation is also foundational to oncogenic transformation, which is observed in low-grade noninvasive pancreatic intraepithelial neoplasms and acinar-to-ductal metaplasia.^124,125^ *KRAS* mutations are thus considered to be an initiating event for this aggressive cancer type.^123^ Sequencing of PDAC exomes revealed a preponderance of *KRAS* oncogene activation along with inactivation of tumor suppressor genes (*CDKN2A*, *TP53*, *SMAD4*, and *BRCA2)*, extensive chromosomal loss, gene amplification, and telomere shortening.^124,126^ While mostly characterized in NSCLC, understanding the impact of co-occurring mutations and alterations, along with KRAS, is important not only for potential prognostication but also for developing potential therapeutic interventions. For example, patients with resected PDAC (n = 587) with the *KRAS G12D* isoform and *TP53* comutation have a better prognosis, as reflected by improved OS and PFS, than those with the co-occurrence of *TP53* with other *KRAS* isoforms (G12V/R or others) (median OS 25.9 vs. 16.9 months; p = 0.038).^127^

CRC is the second most common cancer with *KRAS* mutations.^128^ CRC is also the second leading cause of cancer-related deaths in the United States.^11,129^ Similar to PDAC, KRAS activation is an essential step in the CRC adenoma-to-carcinoma sequence: following the initial mutagenic loss of the adenomatous polyposis coli (*APC*) gene, which results in the loss of intestinal differentiation, activating mutations in *KRAS* and subsequent nuclear translocation of β-catenin are responsible for progression to carcinoma by increasing the growth rate of the adenoma and expansion of the “malignant clone” for further spread and metastasis.^130^ *KRAS* and *BRAF* mutations facilitate the transformation of adenomas into larger tumors. Other mutations in *PIK3CA*, *SMAD4,* and *TP53* facilitate malignant growth with the potential for invasion.^131^ *KRAS* mutations occur in ~40% of cases, and the most common *KRAS* mutation subtypes in CRC are *KRAS*^*G12D*^ and *KRAS*^*G12V*^.^132^ The mutation itself confers resistance to upstream anti-EGFR therapy, hence leading to a worse prognosis.^133^

Lung cancer is the third most common cancer with *KRAS* mutations (~21.20%), with an even higher prevalence (up to 40%) reported in some studies.^74,134^ The most common *KRAS* mutation subtype found in NSCLC (which accounts for 85% of all lung cancers) is the *G12C* mutation (39.11%), followed by variants such as *G12V* and *G12D*, which account for 17.78% and 18.44%, respectively.^135–138^ This likely partially explains the clinical outcomes of lung cancer, which remains the leading cause of cancer-related deaths in the United States,^11^ as point mutations in *G12C* are indicative of a worse outcome than other mutations (*G12V* and *G12D*).^138^ While the specific role of KRAS in carcinogenesis leading to NSCLC is not as clearly defined as it is in PDAC and CRC, multiple oncogenic events and mutations co-occur with KRAS mutations, contributing to resistance through compensatory mechanisms and highlighting the important role of delineating and characterizing those comutations.^139^ In fact, comutations in tumor suppressor genes such as *STK11*, *KEAP1*, *TP53*, *SMARCA4*, and *CDKN2A/CDKN2B* are common in *KRAS*-mutant lung cancers and play key roles in molecular and clinical heterogeneity, such as defining disease subsets on the basis of distinct biology, immune microenvironments, and therapeutic susceptibilities.^136,140^ Compared with nonsmokers, KRAS-mutated cancers in smokers have a complex mutational profile with comutations such as *STK11 (10.3–28.0%)*, *KEAP1 (6.3–23.0%)*, and *TP53 (17.8–50.0%)*.^136,137^ Co-occurring gene mutations, such as those in *STK11* and *KEAP1*, are associated with poor outcomes with chemotherapy.^136^

*KRAS* mutations are infrequent in breast cancer (BCa) and have been reported in certain triple-negative breast cancers (TNBCs).^141^ However, KRAS proteins are still known to be upregulated in BCa (compared with adjacent normal breast tissue), mostly through upstream activation by human epidermal growth factor 2 (HER2) and EGFR, both of which are frequently overexpressed in breast cancer, resulting in amplification of the RAS signaling pathway.^141–143^ Activated, wild-type KRAS also plays an important role in promoting the activation of oncogenic pathways; for example, depletion of the p21WAF1/CIP1 cyclin-dependent kinase inhibitor in BCa exaggerates KRAS signaling, leading to PI3K activation and de novo lipid biogenesis.^144,145^ Finally, one BCa-unique regulatory mechanism of KRAS activation is miRNA-382-5p, which interacts with and regulates the RAS-like estrogen-regulated growth inhibitor (RERG)/RAS/ERK axis, leading to enhanced BCa progression. In TNBC specifically, RAS protein activator like 2 (RASAL2) acts as a GAP gene and activates RAC1, hence promoting TNBC progression.^146^ The activation of the RAS-MAP pathway in TNBC has been linked to immune evasion and increased metastasis.^147^

In addition to mutations in *PIK3CA* and *PTEN*, *KRAS* mutations are the most frequent mutations in gynecological cancers, accounting for 22, 18, and 12% of all mutations, respectively. In ovarian cancer, co-occurring *KRAS* and *BRAF* mutations have been described in low-grade serous ovarian cancer.^148^ In cervical cancer, *KRAS* mutations have been identified as the second most common oncogenic mutation following *PIK3CA*, albeit with a low prevalence (2–4.3%), making it a rare event.^149^ In type I estrogen-related endometrial cancer, *KRAS* mutations occur at a relatively high frequency of 10–30%.^150^ Unlike other cancer types, the exact role of the KRAS pathway in tumorigenesis is not clearly defined and remains investigational, including a potential role in upregulating estrogen receptors and hypermethylation of the *KRAS* promoter as a mechanism of activation in endometrial carcinogenesis.^151–153^

Mutations in *KRAS* have also been detected in other solid tumors, but with a lower frequency and rarer occurrence. *KRAS* mutations account for 7% of prostate cancer (PCa) cases, leading to the activation of downstream effector proteins.^128^ Mutant KRAS plays a transformative role in PCa tumorigenesis by promoting cancer stemness and metastasis to bone, with KRAS rearrangements shown to promote PCa metastatic progression.^153–156^ In melanoma, while mutations in *KRAS* are even rarer, accounting for only 1.7% of cases, inhibiting its downstream effector BRAF is one example of a success story of targeting downstream effectors of KRAS for cancer therapy. Indeed, mutations in *BRAF* are prominent in melanoma, and inhibition of BRAF is a common, standard therapy for the treatment of *BRAF*-mutant melanoma. Recent work has shown that inhibition of wild-type *KRAS* in *BRAF*-mutant melanoma can be a potential strategy to overcome resistance to BRAF inhibition, since inhibition of *KRAS* functions synergistically with BRAF inhibition to reduce proliferation and induce apoptosis independent of *BRAF* mutation status.^157^ *KRAS* mutations are rare in gliomas, including glioblastomas, where alterations in the RTK/PI3K pathway are relatively common drivers.^158^ When present, *KRAS* mutations in gliomas are typically associated with a subset of pediatric low-grade gliomas rather than adult high-grade tumors. *KRAS* mutations are also observed in digestive cancers other than PDAC and CRC: in gastric cancer (~5-10% of gastric adenocarcinomas, mainly in the intestinal subtype), esophageal cancer (more prevalent in adenocarcinoma than in squamous cells, particularly in association with Barrett’s esophagus), biliary tract cancer (often coexisting with FGFR and IDH1/2 mutations), and hepatocellular carcinoma (uncommon, with greater prominence of other pathways, such as Wnt/β-catenin and TP53).^159–163^

The variation in the prevalence, subtypes, and regulation of *KRAS* mutations, as well as the impact of co-occurring mutations, not only reflects differences in disease behavior but is also important when designing therapeutic strategies to target KRAS.

In fact, such differences reflect biochemical heterogeneity, manifested by variations in mutational characteristics such as binding affinity to downstream effectors, response to therapy, and metastatic patterns.^74^ For example, *KRAS* mutations lead to constitutive activation of KRAS proteins either by impeding their interaction with GAPs or by decreasing their intrinsic GTPase activity.^77,161^ The intrinsic GTPase activity of *KRAS*, which allows it to auto-inactivate downstream signal propagation, was highly variable for each KRAS mutant; however, G12C has no impact on the intrinsic inactivation rate, G12D results in a 7-fold decrease in GTPase activity, and G12V and G13D mutations result in an intermediate decrease in the hydrolysis rate. Similarly, with GAP-mediated inactivation/hydrolysis of KRAS, mutations in codons 12 and 13 result in a subdued response to extrinsic GTPases, with most mutants showing a 97–99% decrease in GAP-mediated hydrolysis.^164,165^ *KRAS* mutants with high RAF affinity (WT, G12A, G12C, G13D, and Q61L) and low intrinsic GTPase rates (G12A, G12R, G12V, Q61L, and Q61H) were found to have increased RAF activation and sustained RAF activation compared with mutant forms with low RAF affinity (G12R, G12V, and G12D) and high intrinsic GTPase rates (WT, G12C, G12D, and G13D). KRAS-G12C and G12V preferentially activate the RAL A/B signaling pathway, whereas KRAS-G12D tends to activate the PI3K-AKT-mTOR pathway.^74^ Analyzing the distinct biochemical properties of *KRAS* isoforms and categorization of *KRAS*-driven cancers will help streamline and focus on specific targets of therapy, whether a specific effector, an isoform, or a pathway.

Similarly, understanding co-occurring mutations and intra-driver heterogeneity is crucial for the development of novel therapeutic interventions, namely, combinational approaches. A collection of co-occurring mutations alongside driver mutations plays a significant role in tumor heterogeneity.^140^ These comutations can markedly affect *KRAS* function and influence tumor development and progression.^74^ A study analyzing NSCLC samples (n = 1078) bearing *KRAS* mutations revealed that 53.3% of these patients also had comutation, with *TP53* being the most common (39.4%). Other comutations identified include the serine/threonine kinase 11 gene (*STK11;* 19.8%), Kelch-like ECH-associated protein 1 gene (*KEAP1;* 12.9%), ATM serine/threonine kinase gene (*ATM;* 11.9%), Met amplifications (15.4%) and others, including the rare co-occurrence of *EGFR* (1.2%), which were initially thought of as mutually exclusive with *KRAS* mutations.^6,136,138,165^ These different comutations result in variable impacts. For example, NSCLC tumors with *KRAS/STK11* comutations are associated with a “cold” tumor microenvironment (TME) that has a paucity/exclusion of CD8+ tumor-infiltrating T cells, a preponderance of T-regulatory cells, innate resistance to PD-L1 inhibition, and inferior outcomes with PD-L1 inhibitor therapy and chemoimmunotherapy.^74,136,166^ Tumors with cooccurring *KRAS/TP53* mutations have a TME that is rich in inflammatory cells and dendritic cells. They are also associated with genomic instability, a high tumor mutational burden, and a better response to PD-L1 inhibitors due to increased expression of PD-L1 receptors and shorter OS in patients treated with platinum-based adjuvant chemotherapy.^136^ *KRAS* mutations control the intrinsic features of tumors, such as proliferation and survival, as well as the extrinsic environment that allows them to spread by promoting immune escape. The tumor microenvironment associated with *KRAS* is often inflammatory, which increases the occurrence and development of cancer. *KRAS*-mutant cancers are known to evade the immune response and contort with the TME by upregulating PD-L1 receptor expression, downregulating MHC-I expression on tumor cells, and increasing the secretion of IL-10 and NFκB to induce immunosuppressive T-regulatory cells.^74,167^ Thus, these comutations call for caution when treating KRAS-driven cancers to allow for a personalized therapeutic approach for a better response.^74^ The approval of next-generation sequencing (NGS) by the FDA will help advance care for heterogeneous cancer populations and advance our grasp of the role of comutations and *KRAS* mutation subtypes in disease pathogenesis and outcomes.^138^

### Clinical and prognostic implications of mutated KRAS

Clinically, and as a reflection of the previously described pathways, patients with *KRAS* mutations have a predilection for metastasis to the lung and brain, with G12C and G12D variants being inclined toward bony metastasis and G12V mutants favoring pleuropericardial spread.^74^ The biochemical and molecular heterogeneity described above also reflects differences in the clinical and prognostic implications of mutated *KRAS*, as highlighted below.

In a study of patients with left-sided CRC with microsatellite stability (MSS), KRAS-mutant variants were found mostly in the lung metastatic group, with a lower proportion in the liver metastatic group. Another study of metastatic CRC with mutations between primary and metastatic lesions (progressive and regressive subgroups) revealed a strong association between the *KRAS* regression trajectory (*mut-KRAS*→ *wild-type-KRAS*) and oligometastatic status. The study also revealed a favorable response to chemotherapy in metastatic CRC patients with regressive discordant mutational profiles.^168^ Overall, patients with metastatic *KRAS*-mutant CRC have lower overall survival (OS) and relapse-free survival (RFS).^133^ KRAS status also affects the response to non-KRAS-targeted therapies: metastatic *KRAS*-mutant cancers are known to be resistant to anti-EGFR therapy, leading to a worse prognosis.^133^ *KRAS* mutations increase the risk of CRC metastasis, disease recurrence, and death in patients with CRC.^131^ A study was conducted with 1886 patients who had stage II-III rectal cancer and a history of neoadjuvant chemoradiation (nCRT) and proctectomy with the aim of analyzing the relationships between *mKRAS* and pathologic complete response (pCR), the neoadjuvant rectal (NAR) score, and survival in patients with locally advanced rectal cancer. *mt*-*KRAS* was not associated with pCR or the NAR score; however, *mt-KRAS* was independently associated with a worse prognosis and survival.^169^ In a meta-analysis reviewing the impact of mutations in genes such as *TP53*, *RAS*, *SMAD4*, *PIK3CA*, and *BRAF* and the MSI status on response to treatment and prognosis, in locally advanced rectal cancer (LARC), *KRAS* mutations were associated with a greater risk of nonresponse following preoperative radiation therapy in LARCs (OR = 1.80, 95% CI: 0.39–20.05). Mutated *KRAS* was found to be a detrimental marker for the response of LARCs to preoperative radiation therapy. This association was even more significant in patients who did not receive preoperative cetuximab (OR = 2.17, 95% CI: 1.41–3.33) than in those who did (OR = 0.89, 95% CI: 0.39–20.05).^170^

Similar to CRC, *KRAS* mutations are also markers of a poor prognosis in patients with PDAC, but the same generalization cannot be made for patients with NSCLC, with studies showing controversial results. Compared with patients with wild-type *KRAS*, patients with *KRAS* codon 12 mutations have a dismal prognosis, with patients with *G12C and G12V* mutations having worse overall survival.^132^ Despite the limitation of a small sample size, a study by Safi et al. that included 39 PDAC patients (including both short- and long-term survivors) revealed that patients with *KRAS G12D* mutations had a significantly worse OS than individuals with different pathogenic *KRAS* mutations or other mutations.^171^ Another study involving 110 patients with unresectable PDAC who underwent first-line gemcitabine and nab paclitaxel therapy revealed that wild-type *KRAS* is an independent predictor of improved OS and PFS compared with mutant *KRAS*.^172^

In patients with adenocarcinoma of the lung, compared with wild-type patients, patients with *KRAS* mutations are more likely to be diagnosed at an older age (>45 years) and have an increased rate of metastasis to the brain and liver. A lower KRAS and LKB1 (*STK11*) copy number, along with nodal stage and patient age, predict brain metastasis with good accuracy (area under the ROC curve of 0.832; p < 0.001) in patients with NSCLC.^173^ Despite these observations, however, *KRAS* mutations do not significantly affect the prognosis of patients with NSCLC. A meta-analysis conducted by Yangyang Xu et al. investigating the efficacy of immune checkpoint inhibitor (ICI) therapy in NSCLC patients revealed no association between *KRAS* mutation and the efficacy of ICIs.^174^ Another study evaluating the impact of *KRAS* mutation on the efficacy and safety of ICIs in patients with advanced NSCLC did not find any statistically significant difference in median OS.^175^ A retrospective study of patients with stage IV lung adenocarcinoma treated with first-line pembrolizumab monotherapy revealed a statistically insignificant difference in OS between patients with *mt-KRAS* and *wt-KRAS*, suggesting that KRAS has no prognostic value with respect to treatment with pembrolizumab.^176^

However, other studies have reported differing results, indicating that further investigation is needed.^177,178^ Interestingly, another study involving patients with unresectable stage III NSCLC treated with chemoradiation followed by durvalumab consolidation revealed that *KRAS*-mutant lung cancers derived the greatest benefit from immunotherapy, reflected by their better PFS than did those with *BRAF* and *EGFR* mutations and *ALK* rearrangements.^177^

## Challenges and resistance to currently available KRAS inhibitors

Despite the FDA approval of sotorasib and adagrasib, KRAS inhibitor monotherapy remains noncurative. In the pivotal CodeBreaK 100 trial, which supported sotorasib approval for *KRAS*^*G12C*^-mutant NSCLC, the ORR was ~41%, with a median PFS of 6.3 months and a 2-year OS of ~30%.^179^ Similarly, in KRYSTAL-1, which led to the approval of adagrasib, the ORR was approximately 43%, with a median DoR of 8.5 months, median PFS of 6.5 months, and median OS of 12.6 months.^180^ Thus, while both drugs show clinical benefit, most patients eventually develop resistance, and not all patients respond to single-agent monotherapy. Resistance mechanisms-both intrinsic and acquired-are only partially understood. Additionally, KRAS G12C mutations represent only ~15% of *KRAS*-mutated cancers, leaving most patients, including those with common mutations such as G12D (frequent in CRC and PDAC), without approved targeted therapies.^181^ Another challenge is poor blood-brain barrier (BBB) penetration. This is due to both the molecular structure of current inhibitors and the exclusion of patients with active central nervous system (CNS) disease from early trials, rendering the available data on sotorasib or adagrasib effects on the BBB limited.^182^ Retrospective data suggest that up to 40% of patients with KRASG12C-mutant NSCLC can develop CNS metastasis while on adagrasib, highlighting the high propensity to develop brain metastasis and the need to improve the BBB penetration of these inhibitors.^183^ The preplanned exploratory analysis of patients with previous CNS disease enrolled in CodeBreaK 200 revealed that the time to recurrence was longer with sotorasib than with docetaxel (15.8 vs. 10.5 months; hazard ratio (HR): 0.52, 95% CI: 0.26-1.0).^184^ In the KRYSTAL-12 trial, the intracranial ORR in patients with evaluable CNS disease was improved with adagrasib (40%) compared with docetaxel (11%), with improvement in PFS regardless of CNS disease status.^185^ Despite these encouraging results, improving BBB penetration remains a priority. Future clinical trials should include patients with untreated CNS disease and incorporate serial CNS imaging.

### Primary resistance

Primary resistance to KRAS is hypothesized to result from comutation and *KRAS* mutation.^186^ Understanding these mechanisms is crucial for developing therapeutic strategies and combination approaches. Studies in CRC and NSCLC patients have shown that *KRAS* heterogeneity typically arises after therapy, not at baseline.^187,188^ In a large cohort (8750 pretreated *KRAS*-mutant tumors), only 3.5% had more than one *RAS* mutation; in *KRAS*^*G12C*^-mutant tumors specifically, secondary *RAS* mutations appeared in 3% of the samples.^189^ Cannataro et al. reported no evidence of preexisting resistance mutations in KRAS or its downstream genes prior to KRAS G12C inhibitor therapy.^190^ Nevertheless, the mechanisms of primary resistance remain poorly understood. In contrast, specific resistance mutations to KRASG12D have been identified via mutagenesis screening.^191^

### Acquired resistance due to mutational escape

Resistance to de novo mutations often emerges during treatment under the pressure of KRAS inhibition. Mutations at codons 12, 68, 95, and 96 can disrupt the binding of sotorasib and adagrasib.^87,89^ In vitro experiments using Ba/F3 cells treated with either sotorasib or adagrasib revealed 12 distinct KRAS mutations after treatment, including *KRAS*^*Y96D*^ or *KRAS*^*Y96S*^ mutations, which were shown to confer resistance to both agents, along with increased sensitivity to coinhibition with trametinib (a MEK inhibitor) and an SOS1 inhibitor.^192^ Structural modeling has shown that the Y96D mutation alters the switch-II pocket, preventing drug binding.^193^ Other identified mutations, including secondary G13D, R68M, A59S, and A59T mutations, show differential drug sensitivity, indicating that the two structurally similar compounds cannot be interchangeable.^192^

The heterogeneous acquisition of molecular alterations in patients treated with KRAS inhibitors was also highlighted in the biomarker analysis data from the CodeBreaK 100 trial^194^: at the time of progression following sotorasib monotherapy, 28% and 73% of NSCLC and CRC patients, respectively, acquired new molecular alterations, with RTK pathway alterations being the most common. Secondary RAS alterations were also described, occurring more commonly in patients with CRC (16%) than in those with NSCLC (3%). Other treatment-emergent alterations have also been described, including gene amplifications *(KRAS, MET, MYC, MET*), mutations *(PTEN, IDH1/2, TP53, KRAS, NRAS, NF1, KEAP1*), and deletions (*PTEN, CDKN2A/2B*). Similar patterns have been described by Awad et al., who analyzed baseline and posttreatment samples from 38 NSCLC patients who developed resistance to adagrasib (45%).^195^

Interestingly, the *KEAP1*/*STK11*/*TP53* interplay with the *KRAS* status modulates the response to KRAS inhibitors on the one hand and affects the KRAS inhibitor/ICI combination strategies on the other hand. In a single-arm, phase 2 trial of sotorasib in patients with *KRAS*^*G12C-*^mutated, previously treated NSCLC patients, the ORR was 37.1% (95% CI: 28.6-46.2).^196^ The exploratory analysis of this trial evaluated the impact of cooccurring mutations in *STK11*, *KEAP1*, and *TP53*: the ORR in patients with TP53 mutations was 39%, whereas it was 40% in those with STK11 mutations and 20% in those with KEAP1 mutations. The ORR was greater (50%) in patients with mutated *STK11*/wild-type *KEAP1* and lower in those with comutated *KEAP1/STK11* (23%) and wild-type *STK11*/mutated *KEAP1* (14%). While exploratory, this analysis provided clinical evidence for the impact of these mutations on the response to sotorasib, with specific interest in higher responses in the *STK11*-mutated/*KEAP1*-wild-type subgroup, given prior evidence for inferior outcomes and poorer prognosis for *STK11* and *KEAP1* mutations in NSCLC patients and known primary resistance to PD-1/PD-L1 and docetaxel in patients with *STK11* mutations and *KRAS*-mutated NSCLC.^140,197,198^ These findings identified a subgroup with a potential response and suggested that *STK11*-mutant/*KEAP1*-wild-type status is a biomarker for the response to sotorasib in *KRAS*^*G12C*^-mutant NSCLC^199^. Similar observations have been made with adagrasib, with confirmed ORRs of 40.5%, 28.6%, and 51.4% in patients with *STK11*, *KEAP1*, and *TP53* mutations, respectively, and higher response rates in those with *STK11*-mutant/*KEAP1*-wild-type tumors (44.0%) than in those with *STK11*-wild-type/*KEAP1*-mutant tumors (14.3%).^200^ Similarly, *STK11*, *KEAP1*, and *TP53* resulted in a lower response (compared with wild type) in patients with NSCLC treated with divarasib and IBI351.^201,202^

The interaction between *STK11/KEAP1* and *KRAS* inhibition potentially extends beyond KRAS inhibitor monotherapy to impact combination approaches involving KRAS inhibitors and ICIs. The co-occurrence of the 3 mutations has been postulated to alter the TME, with co-occurrence of the *KRAS* and *STK11* mutations resulting in downregulation of MHC II class expression, inhibition of antigen-presenting cells, T-cell activation, and downregulation of chemokine and chemokine receptor expression, whereas co-occurrence of the *KRAS* and *KEAP1* mutations leads to downregulation of chemokine/receptor expression and downregulation of inflammatory proteins and their receptors.^185^ Recent work has demonstrated that the use of CTLA4 ICIs could abrogate *KEAP1/STK11*-related resistance to anti-PD-1/PD-L1 ICIs, hence providing an alternative combination of anti-CTLA4/KRAS inhibition.^203^

More recent work has further investigated the mechanisms of acquired resistance (reviewed in ref. ^125^). For example, in a *KRAS*^*G12D*^ PDAC mouse model treated with MRTX1133, KRAS inhibition resulted in deep tumor regression, ultimately leading to the emergence of resistance.^204^ These findings reflected the molecular amplification of *KRAS, Yap1, Myc, Cdk6, and Abcb1a/b*, as well as the coevolution of drug resistance transcriptional programs.^204^

### Adaptive resistance

Adaptive resistance refers to the rapid reactivation of the RAS‒MAPK pathway. This occurs when the inhibition of MYC target genes (including RTKs and their ligands) is lost, as initially described with BRAF and MEK inhibitors in melanoma therapy.^205–211^ The precise mechanisms underlying adaptive resistance to KRAS^G12C^ inhibitors remain unclear: whether adaptive resistance is mediated by reactivation of mutant KRAS or activation/recruitment of wild-type KRAS remains a subject of debate.^207–211^ In fact, in vitro studies on *KRAS*^*G12C*^-mutant lung, colon, and pancreatic cancer cell lines have shown that adaptive resistance to sotorasib results from the activation of wild-type NRAS and HRAS, thus laying the groundwork for future drug development strategies that involve the targeting of wild-type *RAS*, which we will describe in a later section.^210^

### Histologic transformation as a resistance mechanism to KRAS inhibitors

Epithelial-to-mesenchymal transition (EMT) refers to the downregulation and upregulation of epithelial and mesenchymal genes, respectively, resulting in increased invasiveness and mobility.^212^ This process has been described in EGFR-mutant NSCLC following treatment with EGFR inhibitors and was shown to mediate resistance to these agents via TGFβ.^213,214^ Similar findings have been described in *KRAS*-mutant cell lines in which the EMT gene signature is induced under therapy with KRAS inhibitors, leading to decreased KRAS dependency.^215^ Gene set enrichment analyses have also demonstrated the role of EMT as a mechanism of resistance to sotorasib, with the activation of the PI3K pathway in EMT-induced *KRAS*^*G12C*^-mutant cell lines via bypass of the IGFR-IRS1 signaling pathway.^216^ These observations support translational work whereby combining sotorasib with a PI3K inhibitor, on the one hand, and with PI3K and SHP2 inhibitors, on the other hand, leads to blockage of AKT activation, as well as AKT and ERK activation, respectively.^216^

Finally, adenocarcinoma-to-squamous transformation has also been reported in approximately 22% of patients (n = 2/9) with *KRAS*^*G12C*^-mutant NSCLC following acquired resistance to adagrasib, similar to what has been described with resistance to EGFR inhibitors.^195,217^ More recent work investigating the adeno-to-squamous transition in patients with the *KRAS*^*G12C*^*/STK11* comutation revealed a poor response to adagrasib and an earlier onset of resistance.^218^ Interestingly, transcriptomic and epigenomic analyses revealed an intermediate high-plastic state marked by the expression of an adeno-to-squamous plasticity signature.^218^ This novel observation highlights the role of epigenetic mechanisms in resistance to KRAS inhibition. While the details of this transition and the role of epigenetics remain elusive, such discoveries open the way for conceptual consideration of future combinational approaches with drug chromatin-modulating therapies, such as DNA methylation inhibitors, for example.^218,219^

**Fig. 4:**
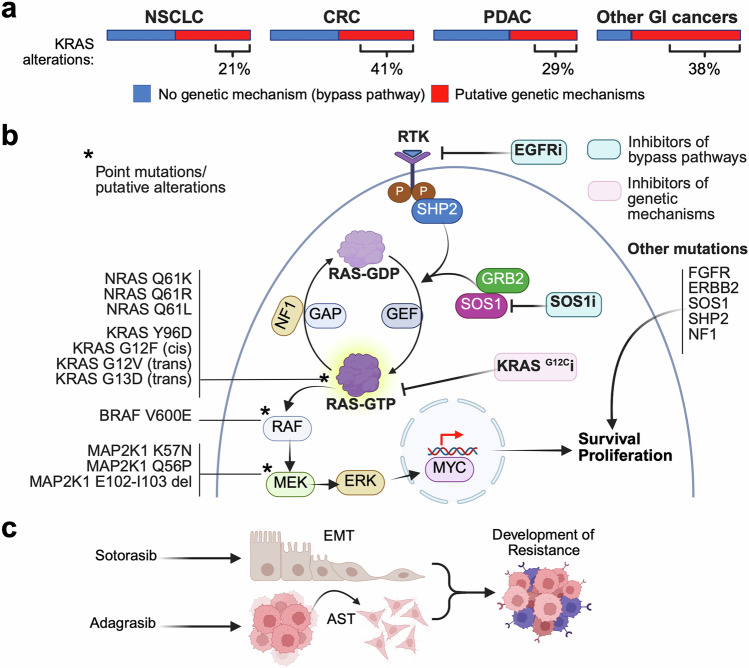
Resistance mechanisms and therapeutic strategies. Resistance mechanisms to KRAS inhibition are classified as putative genetic (point mutations; red) or nongenetic (bypass pathways; blue). **a** Represents the prevalence of each type of resistance mechanism in non-small cell lung cancer (NSCLC), colorectal cancer (CRC), pancreatic ductal adenocarcinoma (PDAC), and other gastrointestinal (GI) malignancies. **b** Represents potential therapeutic strategies to circumvent resistance to KRAS inhibitors, including inhibitors of genetic mechanisms (light pink) and inhibitors of bypass pathways (light blue). **c** Represents histological transformation through epithelial‒mesenchymal transition (EMT) as a mechanism of resistance to KRAS inhibition. Created in BioRender.com

*In summary, while small-molecule inhibitors of* *KRAS*^*G12C*^ *have demonstrated clinical efficacy across solid malignancies and led to regulatory approval for the treatment of NSCLC, there is still an unmet need to develop new strategies to target KRAS that overcome* *KRAS*^*G12C*^ “*off*” *resistance on the one hand and target other common KRAS mutations seen across different malignancies and for whom no approved KRAS inhibitors are currently available on the other hand. We review these ongoing strategies in the upcoming sections*. Figure 4 summarizes resistance mechanisms to KRAS small-molecule inhibitors and highlights potential strategies to overcome them.

## Therapeutic strategies to target KRAS

As discussed earlier, KRAS has long been considered undruggable owing to its structural and molecular features, including the lack of large pockets for drug binding, high affinity of nucleotide binding sites for GTP, elevated intracellular GTP levels, accelerated nucleotide exchange cycles impeding competitive antagonists, and off-target toxicity due to indiscriminate binding to *wild-type KRAS*.^220^ The identification of the new pocket beneath the effector binding switch-II region of KRAS by Shokat et al. allowed the development of small-molecule inhibitors; of those, sotorasib and adagrasib, both G12C inhibitors, are the only two with current FDA approval.^221^ Both are classified as “off” inhibitors given their mechanism of action by direct disruption of the switch-I and switch-II regions, resulting in the arrest of KRAS cycling in its inactive, or “off”, state.^9^ They mediate their action directly by irreversibly binding to the KRAS-GDP state and, to a lesser extent, by decreasing the affinity for GTP.^82,222^ Despite these approvals, the two KRAS^G12C^ inhibitors remain far from curative, with significant ongoing research strategies being investigated to improve responses, overcome resistance to both sotorasib and adagrasib, explore novel strategies other than direct KRAS G12C inhibition, and include other subtypes of mutations. In this section, we review these therapeutic strategies.

### Currently, FDA-approved KRAS inhibitors

Sotorasib is the first clinically available direct KRAS G12C inhibitor that was FDA-approved for the treatment of locally advanced and metastatic NSCLC harboring the *KRAS G12C* mutation.^90^ Adagrasib was later approved as a 2nd-line treatment for metastatic NSCLC with the *KRAS G12C* mutation.^94^ Both sotorasib and adagrasib are KRAS OFF-state small-molecule covalent inhibitors that irreversibly bind to the cysteine residue in the mutant KRAS-G12C switch-II pocket, as described earlier. In preclinical studies using mouse models, sotorasib, in addition to directly inhibiting KRAS, has been found to inhibit the phosphorylation of ERK (extracellular signal-regulated kinase), leading to complete tumor regression in *KRAS*^*G12C*^-mutant cancers.^10,223^

Initial inhuman data from the phase I CodeBreaK 100 (NCT03600883) trial of sotorasib provided evidence for its single-agent activity and efficacy in patients with heavily pretreated (ICIs and platinum-based chemotherapy) advanced solid tumors with *KRAS G12C* mutations, including a partial response (PR) in one of six patients with advanced NSCLC.^82,91,221^ The trial included a total of 129 patients (59 with NSCLS, 42 with CRC, and 28 with other tumors). The NSCLC cohort had an objective response rate (ORR) of 32.2%, with 88.1% achieving disease control (DCR) and a median PFS of 6.8 months. Within the CRC subgroup, the ORR was 7.1%, the DCR was 73.8%, and the median PFS was 4 months. Sotorasib has been shown to have activity against melanoma and pancreatic, endometrial, or appendiceal cancer.^91,221^

In phase 2 of the CodeBreaK trial, which included 124 patients with pretreated advanced NSCLC, sotorasib demonstrated a median OS of 12.5 months and a duration of response (DoR) of 11.1 months. An updated analysis of patients with phase I and II NSCLC revealed a 1-year OS of 50.8% and a 2-year OS of 30.3%. For the PDAC cohort (n = 38), an ORR of 21.1% and a DCR of 84.25% in a heavily pretreated population were noted.^221^ Sotorasib showed sustained clinical benefit across all levels of PD-L1 expression and tumor mutational burden (TMB) and across different comutation profiles. Adverse drug effects were observed in 69.8% of patients, with grade 3 events in 19.8% and grade 4 events in 0.8%.^168^ In a global, open-label phase III trial (NCT04303780; CodeBreaK 200), sotorasib was compared with docetaxel in 345 patients with *KRAS*^*G12C*^ advanced NSCLC who progressed after platinum-based chemotherapy and an ICI.^224,225^ Patients were randomized 1:1 to receive oral sotorasib (960 mg daily) or intravenous docetaxel (75 mg/m^2^, every 3 weeks). At a median follow-up of 17.7 months, the study met its primary endpoint, with a statistically significant improvement in PFS with sotorasib vs. docetaxel (HR: 0.66; 95% CI: 0.51-0.86; p = 0.002). The ORR was also significantly improved (28.1% for sotorasib vs. 12.2% for chemotherapy; p < 0.001). OS, for which the study was not powered, was not statistically significant.^224^ Despite meeting its primary endpoint, the study design did receive criticism, mainly owing to the choice of a suboptimal control arm (docetaxel monotherapy rather than docetaxel/ramucirumab, the approved regimen shown to be superior to docetaxel), as well as the choice of OS as a secondary endpoint.

The Krystal-1 trial (NCT03785249) demonstrated the efficacy of adagrasib in *KRAS*-mutated cancers, with no new safety concerns in the phase II portion of the trial. In 116 patients with pretreated NSCLC, the ORR was 42.9%, with a median DoR of 8.5 months, a median PFS of 6.5 months, and a median OS of 12.6 months. The estimated one-year OS was 50.8%. Adagrasib had a 33.3% ORR in patients with stable intracranial metastasis who had previously received chemotherapy and immunotherapy.^200,221^ Grade 1 and 2 adverse effects were noted in 52.6% of the patients, and grade 3 or higher adverse effects occurred in 44.8% of the patients.^200^ The PDAC subgroup had a 50% ORR and 100% DCR. Non-CRC gastrointestinal *KRAS G12C* cancers (n = 17) had an ORR of 33.5%. For *KRAS G12C*-mutant CRCs (n = 45), the ORR was 22%, with a DCR of 87%, a median DoR of 4.2 months, and a median PFS of 5.6 months.^221^ Compared with docetaxel, adagrasib was evaluated more recently in a phase III trial (KRYSTAL-12) in 301 patients with *KRAS*^*G12C*^ advanced NSCLC who progressed after platinum-based chemotherapy and an ICI.^226^ In this trial, patients were randomized 2:1 (to receive adagrasib), and at a median follow-up time of 9.4 months, the primary endpoint PFS was significantly improved (HR: 0.59; 95% CI: 0.45–0.76; p < 0.0001), with a median PFS of 5.49 vs. 3.84 months. The ORR was also significantly greater with adagrasib (31.9% vs. 9.2%).

Both sotorasib and adagrasib are taken orally, metabolized via CYP3A and CYP3A4, respectively, and excreted predominantly through the fecal route. Adagrasib results in a concentration-dependent increase in the QTc interval and may precipitate ventricular arrhythmias, and no such association is observed with sotorasib.^10^

### Ongoing research and novel therapeutic KRAS-targeting strategies

Following the approval of sotorasib and adagrasib, the field of KRAS research has continued to experience exponential growth, particularly in the exploration of novel strategies.

#### Novel direct KRAS G12C Inhibitors

Other KRAS-G12C inhibitors aimed at improving the potency and selectivity of inhibition are under development. These are listed below. Table 1 contains additional KRAS G12C inhibitors along with their efficacy metrics.

i. *Divarasib (GDC-6036)* is 5–20 times more potent and 50 times more selective than either adagrasib or sotorasib. A phase 1 clinical trial (NCT04449874) with 137 patients (60 with NSCLC, 55 with CRC, and 22 with other tumors) reported an ORR and a median PFS of 53% and 13 months, respectively, for the NSCLC cohort. The median DoR was 14 months. The data for the CRC subgroup revealed a confirmed response in 29.1% of patients, with a median PFS of 5.6 months.^202,227^ It is being tested as a standalone drug and in combination with other medications, such as monoclonal antibodies, kinase inhibitors, and SHP2 inhibitors, for patients with advanced KRAS G12C-mutant solid cancers.^221^ The most common adverse effects include nausea (74%), diarrhea (61%), and vomiting (58%).^175^
ii. *Opnurasib* *(JDQ443)*: In vitro studies have revealed promising antitumor activity against *KRAS G12C*-mutant cancers with comutations that are responsible for acquired resistance to sotorasib and adagrasib monotherapy.^221,227^ The KontRASt-01 (NCT04699188) phase Ib/II, involving patients with *KRAS* mutations who had received standard chemotherapy and no prior KRAS inhibitors, had an ORR of 57%. However, 68% of the patients developed adverse effects, including fatigue, nausea, peripheral edema, GI distress, and neutropenia. In the dose expansion part of the KontRASt-01 trial, combination therapies with a PD-1 antibody or SHP2 inhibitor are being evaluated. KontRASt-03 (NCT05358249) is a combination of various inhibitors, including MEK, CDK4/6, and EGFR. KontRASt-02 (NCT05132075) was started in 2002 as a confirmatory phase III trial evaluating opnurasib monotherapy compared with docetaxel in patients with previously treated locally advanced and metastatic NSCLC before being halted when the manufacturer discontinued the development of opnurasib.^140,174^
iii. MK-1084: The phase 1 KANDLELIT-001 trial evaluated the safety and preliminary antitumor activity of MK-1084, a next-generation, selective KRAS G12C covalent inhibitor in KRAS G12C-mutant CRC and NSCLC tumors, as a monotherapy or in combination with cetuximab or chemotherapy (FOLFLOX).^226^ While the trial is still ongoing, preliminary data suggest that MK-1084 monotherapy, or in combination with cetuximab and/or chemotherapy, has a manageable safety profile with evidence of antitumor activity.

**Table 1:** Summary of phase I/II clinical trials for KRAS^G12C^ inhibitors, reporting key efficacy metrics

KRAS G12C Inhibitor	Trial	Phase	N	ORR (%)	Median PFS (months)	Median OS (months)	DCR (%)	Refs.
Divarasib (GDC-6036)	NCT04449874	1	137	53 (NSCLC) 29.1 (CRC)	13 (NSCLC) 5.6 (CRC)	-	-	^93,202^
Opnurasib (JDQ443)	NCT04699188	1b/2	84	57%	-	-	-	^93,221^
Olomorosib (LY3537982)	NCT04956640	1/2	146	40	9	-	90	^414^
MK-1084	NCT05067283	1	54	22	-	-	-	^415^
Garsorasib (D-1553)	NCT05383898	2	123	50	-	-	-	^416^
Glesirasib (JAB-21822)	NCT05009329	2	119	47.9	8.2	13.6	86.3	^201,417^
Fulzerasib (IBI351)	NCT05005234	2	116	49.1	9.7	NR	90.5	^418^
BI 1823911	NCT04973163	1	17	18	-	-	65	^419^
ZG19018	ChiCTR20220296	1	14	16.7	-	-	66.7	^420^
D3S-001	NCT05410145	1/2	41	75.8	-	-	-	^421^
GEC255	NCT05768321	1b	16	76.9	-	-	92.3	^422^

*ORR* objective response rate, *PFS* progression-free survival, *OS* overall survival, *DCR* disease control rate, *Refs* references, *NR* not reached, *NSCLC* non-small cell lung carcinoma, *CRC* colorectal cancer

#### KRAS G12D inhibitors

*KRAS G12D* mutations are found in CRC and PDAC. The development of G12D-specific inhibitors is more challenging because of the lack of a nucleophilic cysteine residue, making this mutation variant a poor candidate for covalent attack.^227,228^ Using adagrasib as the structural backbone, various modifications led to the development of drugs that bind to the aspartic acid residue in the mutant gene via a salt bridge in the switch-II pocket of KRAS G12D. Fine-tuning of these molecules eventually resulted in the discovery of MRTX1133 by Mirati’s research team. MRTX1133 is a selective noncovalent G12D that can bind reversibly to the inactive GDP-bound state of KRAS G12D and inhibit RAS-RAF effector binding to the active GTP-bound KRAS G12D.^223,228–230^ Additionally, MRTX1133 upregulates the expression and activation of *EGFR* and *HER2* in *KRAS G12D*-mutant mouse models and in human pancreatic cell lines.^230^ MRTX1133 is 1000 times more selective for KRAS G12D than the wild type. This was demonstrated by in-cell western blot assays, which revealed decreased ERK signaling in 24 out of 25 cell lines harboring G12D mutations following treatment with MRTX1133. This finding was further corroborated by Western blot and CellTiter-Glo assays of HPAC cell lines.^229^ It is known to induce long-term tumor regression in immunocompetent mice with established disease. NCT05737706, a phase 1/2 trial assessing MRTX1133 in patients with advanced solid tumors, is ongoing.^227^ MRTX1133 treatment induces changes in the TME, notably an increase in the number of CD8-positive effector T cells, a decrease in the number of neutrophils and myeloid cells, and the reprogramming of cancer-associated fibroblasts (CAFs) and the overall TME. However, these effects are not observed in an immunodeficient state. Tumor regression has been demonstrated in syngeneic (allograft) immunocompetent mouse models but not in immunocompromised mice, indicating that the presence of T cells is needed for the full antitumor effect of MRTX1133.^230^

HRS-4642 is another highly selective KRAS G12D inhibitor that was studied in phase I of the NCT05533463 trial, which included 18 patients with advanced tumors with *G12D mutations* (10 with NSCLC). In total, six patients (33.3%) presented with grade 3 side effects, including anemia, increased lipase levels, and hypercholesterolemia. Preliminary analysis revealed target lesion shrinkage in six patients and stable disease in 11 patients.^227^

TH-Z835, a representative drug developed through a medicinal chemistry approach,^228^ forms a salt bridge with the aspartic acid (ASP) residue, resulting in KRAS G12D inhibition in both active and inactive forms, with preferential binding to either state. The antiproliferative effects of TH-Z835 were demonstrated in both PANC-1 and KPC cell lines harboring *KRAS G12D* mutations. In other non-G12D cell lines, TH-Z835 had antiproliferative effects on 4T1 (KRAS- WT), MIA PaCa-2 (KRAS(G12C)), CFPAC-1 (KRAS G12V), and HCT116 (KRAS G13D) cells. This was associated with decreased pERK and pAKT cellular levels and the induction of apoptosis, leading to off-target toxicity.^231^

GFH375 is an orally bioavailable, highly selective, and potent KRAS G12D inhibitor that targets mutated KRAS in the GTP- and GDP-bound states. Recently, published results from a phase I/II study (NCT06500676) that included patients with KRAS G12D-mutant advanced solid tumors (11 with PDAC, 11 with NSCLC, 5 with CRC and 5 others) were used to evaluate the safety, tolerability and efficacy of GFH375.^232^ The study revealed no dose-limiting toxicities at various dose levels, with good tolerability and promising antitumor activity (ORR: 27.3%, including 3 PR and 4 SD).

Notably, while there are no G12V-directed inhibitors, other strategies, including an mRNA vaccine combined with an ICI and adoptive T-cell therapy, have been described.^233,234^

#### Combination strategies to increase KRAS activity

Several combinational strategies have been explored, considering the previously discussed eventual resistance to KRAS inhibition. On the one hand, these combinations aim to overcome adaptive resistance to currently available inhibitors, and on the other hand, they also enhance and prolong responses by harnessing other cellular and immunologic antitumor processes.

Resistance is mediated through the upregulation and activation of several upstream RTKs, and their ligands have prompted the investigation of “vertical” combinations that target these upstream pathways. Different RTK‒ligand combinations can selectively predominate in specific tumor types. For example, unlike in PDAC, KRAS^G12C^ inhibition in NSCLC and CRC tumors leads to upstream accumulation of activated EGFR and other ERBB receptor family members, eventually leading to escape from the KRAS^G12C^ inhibitor.^235–237^ Accordingly, the coinhibition of upstream and/or downstream activators has been heavily explored.

##### KRAS-EGFR combined inhibition

In KRYSTAL-1 (NCT03785249) and KRYSTAL-10 (NCT04793958), adagrasib was compared with cetuximab, an anti-EGFR monoclonal antibody, in patients with metastatic *KRAS*^*G12C*^-mutated CRC. Data from KRYSTAL-1 involving 94 patients were recently published and revealed that the primary endpoint, ORR, was 34%, and the DCR was 85.1%, with a median DoR of 5.8 months. The median PFS and OS were 6.9 months and 15.9 months, respectively, and treatment-related adverse events of Grade 3 or higher occurred in 27.7% of patients, with none leading to discontinuation and no treatment-related deaths.^238^ The phase 3 KRYSTAL-10 trial is currently ongoing to compare adagrasib monotherapy with the combination of adagrasib and cetuximab in previously treated patients with advanced *KRAS*^*G12C*^-mutated CRC.^239^ KRYSTAL-1 is also investigating the combination of adagrasib with afatinib, a small-molecule EGFR inhibitor, but no data have been published at the time of this review. GDC-6036 (divarasib), another KRAS^G12C^ inhibitor, is also being tested in combination with cetuximab, as well as with erlotinib, an EGFR tyrosine kinase inhibitor (NCT04449874).

Sotorasib was also evaluated in combination with EGFR/HER2 inhibitors. In CodeBreak 101 (NCT04185883), sotorasib is combined with afatinib or panitumumab, an anti-EGFR monoclonal antibody (with or without FOLFIRI),^240^ in patients with NSCLC. The sotorasib/afatinib combination showed a manageable safety profile, as well as an efficacy signal (ORR: 20 and 34.8% in the two-dose cohorts; DCRs of 70% and 73.9%).^241^ Similarly, BI-1810631 (zongertinib), a highly potent, wild-type EGFR-sparing, covalent HER2 inhibitor, was evaluated in combination with KRAS G12C inhibitors in *KRAS*^*G12C*^-dependent NSCLC patient-derived xenograft models.^242^ In this preclinical work, zongertinib exhibited synergistic growth inhibition with both sotorasib and adagrasib, induced persistent tumor shrinkage (not achieved by either KRAS inhibitor alone), and importantly, had no significant added toxicity. Zongertinib was evaluated in a phase I/II study (NCT04886804), with a phase III confirmatory study underway (NCT04886804).

##### KRAS-SHP2 coinhibition

SHP2 is a nonreceptor tyrosine kinase phosphatase that acts as an upstream positive regulator of the RAS-MAPK pathway,^243^ along with modulating other tumor-related pathways and immune cell signaling.^207,244–246^ It thus plays a pivotal role in tumorigenesis, as proven in several models of *KRAS*-mutant NSCLC.^116,247^ Accordingly, many SHP2 inhibitors have undergone preclinical development, showing single-agent activity against the cycling of KRASG12C mutants via the inhibition of GDP-GTP exchange,^207,244^ and some have entered the clinical trial arena. SHP2 GEF activity is downstream of RTKs but upstream of RAS, thus making it an attractive target for preventing and overcoming adaptive resistance to KRAS^G12C^ inhibitors via the inhibition of other signaling pathways, such as the PI3K-AKT signaling pathway.^207,210,211,248^

Coinhibition of KRAS and SHP2 is currently being evaluated in clinical trials. TNO155, an SHP2 inhibitor in combination with adagrasib, is under evaluation in patients with *KRAS*^*G12C*^-mutant solid tumors in the KRYSTAL-2 trial (NCT04330664), with no published results to date.^249^ The same SHP2 inhibitor is also under evaluation in combination with sotorasib (CodeBreak 101; NCT04185883), as well as with JDQ443, a potent KRAS^G12C^ inhibitor, in KontRASt-01 (NCT04699188). CodeBreaK 101 also tests the combination of sotorasib with RMC-4360 (a SHP2 inhibitor).^240^ Table 2 includes other ongoing clinical trials involving different combinations of KRAS and SHP2 inhibitors.

**Table 2:** Currently ongoing trials involving various strategies to target KRAS

Targeting strategy	Target	Drug	Phase	Trial identifier(s)	Comments
***KRAS OFF(GDP)-state inhibition***	KRAS^G12C^	Sotorasib (AMG 510)	Approved	NCT03600883, NCT04185883, NCT04303780, NCT04933695, NCT04625647, NCT05398094, NCT05074810, NCT04380753, NCT05311709, NCT05054725, NCT05400577, NCT05180422, NCT04667234, NCT05198934, NCT05374538, NCT05313009, NCT05118854, NCT05273047, NCT05251038, NCT04892017, NCT04959981, NCT04720976	Approved for previously treated advanced-stage KRAS^G12C^-mutant NSCLC. Multiple ongoing trials evaluating various combinations
		Adagrasib (MRTX849)	Approved	NCT03785249, NCT04793958	Approved for (i) previously treated advanced-stage KRAS^G12C^-mutant NSCLC, and (ii) combined with cetuximab for previously treated advanced-stage KRAS^G12C^-mutant CRC.
		GDC-6036 (RG6330)	Phase 3	NCT04449874	Tested alone and in combination with various monoclonal antibodies, kinase inhibitors, and the SHP2 inhibitor GDC-1971.
		JDQ443	Phase 3	NCT04699188, NCT05132075, NCT05358249	Tested as monotherapy and in combination with TN0155 (SHP2 inhibitor) +/ tislelizumab (anti-PD-1). KontRaSt-02: in combination with docetaxel KontRaSt-03: platform study for various JDQ443-based combinations.
		LY3537982	Phase 1	NCT04956640	Tested in KRAS^G12C^-mutant solid tumors, alone and in combination with various other agents.
		D-1553	Phase 1/2	NCT04585035, NCT05383898, NCT05379946	
		JNJ-74699157	Discontinued	NCT04006301	
		BI 1823911	Phase 1/2	NCT04973163	
		JAB-21822	Phase 1/2	NCT05009329, NCT05194995, NCT05002270, NCT05276726, NCT05288205	
		MK-1084	Phase 1/2	NCT05067283	
		GFH925	Phase 1/2	NCT05005234; NCT05497336	
		YL-15293	Phase 1/2	NCT05119933	
		HS-10370	Phase 1/2	NCT05367778	
		BPI-0421286	Phase 1	NCT05315180	
		GH35	Phase 1	NCT05010694	
		GEC255	Phase 1	CTR2021486	
		D3S-001	Phase 1	NCT05410145	
		HBI-2438	Phase 1	NCT05485974	
		SY-5933	Phase 1	NCT06006793	
	KRAS^G12D^	MRTX1133	Phase 2	NCT05737706	In KRAS^G12D^ patients with advanced solid tumors.
		HRS-4642	Phase 1	NCT05533463	
		INCB161731	Phase 1	NCT06179160	
		QTX3046	Preclinical		
		VRTX153	Preclinical		
		ERAS-4	Preclinical		
		LY3962673	Preclinical		
		JAB-22000	Preclinical		
	KRAS^G12V^	JAB-23000	Preclinical		
	KRAS^multi^	JAB-2340	Preclinical		Targets G12D, V, A, R, G13D, and Q61H variants; inhibits both GTP and GDP-bound KRAS
		BBP-454	Preclinical		Targets G12, G13 and G61 variants
***KRAS ON(GTP)-state inhibition***	KRAS^G12C^	RMC-018	Preclinical		
		RMC-6291	Phase 1	NCT05462717	ORR: 57% (NSCLC), 44% (CRC)
	KRAS^G12V^	RMC-9805 (RM-036)	Phase 1	NCT06040541	Ongoing; monotherapy or in combination with RMC-6236
	KRAS^G13C^	RMC-8839	Preclinical		
	Pan-RAS	RMC-6236	Phase 1/1b	NCT05379985	First-in-class; RASolute-302, phase III trial is planned (NCT06625320)
		RMC-7997	Preclinical		
***SHP2 inhibition***	SHP2	GDC-1971	Phase 1	NCT04449874	In combination with KRAS^G12C^ inhibitor GDC-6036
		TNO155	Phase 1/2	NCT04699188	Combined with KRAS^G12C^ inhibitor JDQ443 and/or tislelizumab (anti-PD-1)
		RMC-4630	Phase 1	NCT03634982, NCT05054725, NCT04418661, NCT03989115, NCT04916236, NCT04185883	
		JAB-3068	Phase 1	NCT03518554, NCT03565003, NCT04721223	
		JAB-3312	Phase 1	NCT04045496, NCT04121286, NCT04720976, NCT05288205	
		SHP099	Preclinical		
		RMC-4550	Preclinical		
***SOS1 inhibition***	SOS1	BI 1701963	Phase 1	NCT04973163, NCT04111458, NCT04975256, NCT04835714, NCT04627142	
		BAY-293	Preclinical		
		BI-3406	Preclinical		
		RMC-5845	Preclinical		
***Vaccines/nucleic acid-based***	Pan-RAS	Long-peptide vaccine	Phase 1	NCT04117087	Targets G12C, G12V, G12D, G12A, G13D, and G12R variants of KRAS.
		mDC3/8-KRAS vaccine	Phase 1	NCT03592888	Targets G12C, G12V, G12D, or G12R; dendritic cell-based vaccine
		mRNA-5671(V941)	Phase 1	NCT03948763	Targets G12C, G12D, G12V; tested as monotherapy and in combination with pembrolizumab.
	KRAS^G12D/12R^	ELI-002 2P	Phase 1	NCT04853017	Amph-modified G12D-mutant and G12R-mutant KRAS peptides with an immunostimulatory Amph-CpG-790.
	KRAS^G12D^	siG12D KRAS_LODER	Phase 1/2	NCT01188785, NCT01676259	
		siG12D-loaded iExosomes	Phase 1	NCT03608631	
***Adoptive T-Cell therapy***	KRAS^G12V^	Specific TCRs	Phase 1/2	NCT04146298	
***PROTACs***	KRAS^G12C^	LC-2	Preclinical		Combination of adagrasib and VHL, a ligand for the E3 ligase
	KRAS^G12D^	ASP3082	Phase 1	NCT05382559	KRAS^G12D^-targeted degrader
	Pan-RAS	K27 SPOP	Preclinical		Targets G12C, G12D, G12V and Q61H

The table groups the trials by targeting strategy and target, provides information on its clinical development stage (approved, phase 1, 2, or 3, or preclinical development), trial identifier, and relevant comments

##### Coinhibition of KRAS and MEK

Preclinical work has provided evidence that *KRAS*-mutant tumor cells are inherently resistant to MEK inhibitors secondary to RAF-mediated MEK activation, hence providing a rationale for the simultaneous inhibition of mutant KRAS and MEK.^250,251^ The CodeBreaK 101 trial evaluated trametinib in combination with sotorasib, with or without panitumumab, in patients with various *KRAS*^*G12C*^-mutant solid tumors (NCT04185883). Preliminary data (n = 41 patients; 18 with NSCLC and 18 with CRC) revealed the safety and tolerability of the combination, with mostly grade ≤2 toxicities (rash, diarrhea, nausea, and vomiting).^252^ In terms of efficacy, the combination was associated with clinical benefit in patients with NSCLC (15/18 achieved disease control, including 3 PRs) and CRC (15/18 achieved disease control, including 2 PRs).^252^ More importantly, the benefit was observed even in patients who experienced prior disease progression with a KRAS^G12C^ inhibitor.

##### Targeting SOS1

SOS1/SOS2 are RAS-GEFs with crucial roles in regulating KRAS activation, and SOS1/2 inhibition is another strategy for suppressing RAS mutants that retain some activity, despite RAS inhibition. SOS1 activation has been shown to be a key mechanism of adaptive resistance to both KRAS and MEK inhibitors.

BI-3406 is an orally available, SOS1-specific small-molecule inhibitor that binds and disrupts the interaction between SOS1 and RAS GDP. Given the known effect of SOS1 on MEK, BI-3406 has been evaluated in combination with trametinib in *KRAS*^*G12C*^-mutant and *KRAS*^*G13D*^-mutant cell lines, resulting in synergistic growth inhibition. Since these inhibitors target the SOS1-KRAS interaction, their effects include different mutation variants (G12C, G12V, G12S, G12A, and G12D), which have translated, preclinically, into decreased cellular proliferation across a wide range of *KRAS*-mutant tumors, notably NSCLC, PDAC, and CRC cell lines.^253^ In *SOS2*-knockout mouse models, BI-3406 was more effective at decreasing RAS-GTP levels and arresting cellular proliferation, suggesting that SOS2 activity is a potential resistance mechanism of KRAS inhibition.

Like BI-3406, BI 1701963 also targets and impairs KRAS-SOS1 binding, with preclinical evidence of the inhibition of KRAS-activating mutations.^254^ Currently, BI 1701963 is being evaluated in early-phase trials, either alone (NCT04111458) or in combination with adagrasib (KRYSTAL-14; NCT04975256). Additionally, BI 1701963 is being evaluated with trametinib in a phase I trial (NCT04111458) on the basis of the same mechanistic rationale described earlier. BAY-293 is another SOS1 inhibitor that was shown to exhibit activity against *KRAS*^*G12C*^-mutant cell lines in combination with ARS-853, a KRAS^G12C^ inhibitor.^255^ BAY-293 has also demonstrated cytotoxicity against other *KRAS*-mutant NSCLC and PDAC cell lines and synergy with MEK, CDK4/6, topoisomerase I, or EGFR inhibitors.^256^

Whether the combination of KRAS/SOS1 inhibitors is more rational than the combination of KRAS/SHP2 inhibitors warrants careful consideration^120^: while SOS1 inhibitors are selective (they do not affect SOS2), SHP2 is required for full activation of the SOS1/2 complex, and SHP2 inhibition would hence induce inhibition of the full complex and may be superior to SOS1 inhibition alone. However, unlike SHP2 inhibitors, which indirectly affect KRAS, SOS1 inhibitors directly target RAS GDP/GTP exchange and may thus be less susceptible to bypass mechanisms. In addition, SHP2 inhibition has an indirect beneficial effect through the modulation of the TME (while SOS1 inhibition does not), which we discuss later. Until direct studies comparing SHP2 and SOS1 inhibitors are performed, with subsequent clinical evaluation, the comparison remains a theoretical concern.

##### KRAS and XPO1 inhibition

The nuclear export protein exportin 1 (XPO1) acts downstream and at the convergence of many cancer signaling pathways.^257^ As a nuclear exporter, it plays a vital role in maintaining cellular homeostasis by controlling the export of protein cargos from the nucleus to the cytosol.^257^ In cancer, the export of tumor suppressor genes from the nucleus prevents them from performing their normal function in regulating cell growth^258^; hence, increased XPO1 expression has been correlated with poor prognosis across several solid and hematologic malignancies. In *KRAS*-mutant cancer cells, published evidence reveals a dependency on XPO1-mediated nuclear transformation for growth and survival.^259^ Similarly, evidence from *KRAS*-mutant lung cancer patient-derived xenografts revealed the antitumor efficacy of the XPO1 inhibitor selinexor.^260^ Moreover, XPO1 expression is linked to resistance to various therapies, making it a promising target for novel cancer therapies.^261–263^

Our group previously published evidence from a large cohort of NSCLC patients (n = 18,218), 19% of whom had *KRAS* comutations, which revealed that the presence of XPO1 pathogenic mutations, but not amplifications, was associated with poor survival in patients with NSCLC.^263^ This association was maintained even when samples harboring driver mutations were excluded, and in a subsequent study involving KRAS^G12C^ inhibitor-resistant cell lines, the addition of selinexor to a KRAS^G12C^ inhibitor resulted in increased anticancer activity in both in vitro and in vivo preclinical models.^264^ These data suggest that combining selinexor with a KRAS^G12C^ inhibitor may have synergistic efficacy in patients who have developed resistance to therapy with a KRAS inhibitor, and various clinical trials are currently ongoing to test such combinations (Table 2). In a recent phase 1–2 trial, Selinexor was tested in combination with docetaxel in previously treated, advanced *KRAS*-mutant NSCLC patients (n = 40): of 32 patients evaluated for efficacy, 7 (22%) had a PR, and 18 (56%) had an SD, with a median PFS of 4.1 months and a median OS of 7.1 months, with no difference based on the various KRAS mutation types.^265^ The XPO1 inhibitor selinexor is currently approved in relapsed/refractory (R/R) multiple myeloma in combination with bortezomib and dexamethasone and as a monotherapy for patients with R/R diffuse large B-cell lymphoma.^266,267^

##### KRAS and PAK4

p21-Activated kinase (PAK) serine/threonine kinases are important effectors of the small GTPases Rac and Cdc42 and therefore play a major role in modulating changes in actin cytoskeleton organization and dynamics.^268^

PAK4 has been implicated in several specific cellular processes critical for oncogenic transformation, including blocking apoptosis and programmed cell death, inhibiting cell adhesion, promoting cell migration, and promoting anchorage-independent growth.^268–271^ Notably, PAK4, as opposed to other PAK isoforms, can transform normal cells.^272^ PAK4 upregulation has been identified across several human cancer cell lines and patient samples, with amplifications of the chromosome region encoding PAK4, which is frequently observed in PDAC, CRC, and ovarian cancer.^273,274^ Overexpression of PAK4 was shown to transform NIH-3T3 cells via oncogenic Ras-dependent transformation and increased anchorage-independent growth and tumor formation in nude mice.^271^ Similarly, PAK4 knockdown has been shown to have an opposing effect on cell transformation.^275^ Targeted shRNA-mediated knockdown of PAK4 inhibited the migration, invasion, and proliferation of ovarian cell lines with increased nuclear and cytoplasmic expression of PAK4 and pPAK4.^274^

With respect to KRAS, PAKs are activated in KRAS^G12C^ inhibitor-resistant cancer cells, and PAK4 mediates this resistance through the activation of both the MAPK pathway and the PI3K pathway.^276^ The critical role of PAK4 in mediating resistance to therapy has also been described in gastric and cervical cancers resistant to chemotherapy.^277^ Targeting PAK4 was further shown to inhibit Ras-mediated oncogenic signaling, hence making it an attractive partner for coinhibition with KRAS. KPT9274 is a small-molecule inhibitor that targets PAK4 and has been studied as a potential cancer therapy in both preclinical and early-stage clinical trials.^278^ Our group further demonstrated that KPT9274, in combination with KRAS^G12C^ inhibitors, has synergistic effects and enhances the anticancer activity of KRAS inhibitors in RAS^G12C^ inhibitor-resistant cancer models.^279^ These findings thus lay the groundwork for clinical studies investigating the effectiveness and safety of combining KPT9274 with sotorasib in patients who have not responded to sotorasib monotherapy.

While the previously discussed combinational strategies have focused on targeting upstream/downstream effectors that mediate resistance to KRAS inhibitors, alternative strategies have been proposed to overcome this adaptive resistance. These include cotargeting of cell cycle regulators, the mTOR pathway, and “stemness” gene transcriptional regulation.

##### Coinhibition of cell cycle regulators

Cell cycle inhibitors such as abemaciclib and ribociclib, which impair cell cycle progression, target convergent nodes of the RAS-MAPK and PI3K-AKT pathways and provide a potential combination strategy to synergize with KRAS inhibitors.^280^ Preclinical models, both in vitro and in vivo, have suggested a synergistic benefit from the coinhibition of CDK4/6 and KRAS^G12C^ in both NSCLC and PDAC.^281^ This concept was clinically tested in CodeBreak 101 by combining sotorasib and palbociclib (NCT04185883).

Like CDK4 and CDK6, Aurora kinases (*AURKA*, *AURKB*, and *AURKC*) play crucial roles in cell cycle regulation and progression to mitosis.^282^ In NSCLC, *AURKA* expression was found to be a poor prognostic factor, and Aurora kinase signaling upregulation has been implicated in KRAS-mutant malignancies, namely, mutant KRAS NSCLC, and in sotorasib-resistant cell lines.^283,284^ In these cell lines, the knockdown of *AURKA* or AURKA/AURKB inhibition resulted in antitumor activity. In preclinical models, combined inhibition of AURKA and KRAS^G12C^ via sotorasib and VIC-1911 (a selective AURKA inhibitor) has shown synergistic effects and increased activity in NSCLC cell lines intrinsically resistant to sotorasib.^284^ WEE1 is another cell cycle checkpoint regulator that has been shown to mediate sorafenib insensitivity/poor activity in *KRAS*^*G12V*^-mutant cell lines.^285^ Accordingly, preclinical work in sotorasib-resistant NSCLC cells in vitro and in vivo evaluated the combination of VIC-1911 with adavosertib, which has potential synergistic activity and potential therapeutic utility.^285^

##### Targeting parallel pathways: the AKT-mTOR pathway

The inhibition of mTOR has also been explored in several preclinical PDAC models with resistance to KRAS and MEK inhibition on the basis of the rationale that mTOR inhibition would impair the bypass signaling mediated via phosphorylated Rictor, a component of the mTOR complex 2.^286^ In these PDAC mouse models, coinhibition of KRAS^G12C^ or MEK and mTORC1/2 synergistically impaired ERK and AKT activation, resulting in durable inhibition of growth and metastasis.^286^ The combination of everolimus, an mTOR inhibitor, with sotorasib is being evaluated in CodeBreak 101 (NCT04185883). A recent study using genetically engineered KRAS^G12C^ and KRAS^G12D^ mouse models compared the process and tumor development in these two models.^287^ The molecular changes associated with tumor initiation and adaptation were investigated via RNA sequencing to determine potential therapeutic vulnerabilities in each of the models. This study revealed that, compared with KRAS^G12C^ mice, KRAS^G12D^ mice had more rapid lung tumor formation and reduced survival, and this increased potency was associated with increased PI3K-AKT-mTOR signaling. In addition, KRAS^G12D^ inhibition was enhanced by AKT inhibition both in vitro and in vivo, highlighting the value of cotargeting KRAS and the AKT-mTOR pathway in appropriately selected patients.

##### Coinhibition of transcriptional regulators

Finally, the interaction between YAP (Yes-associated protein) and TEAD (transcriptionally enhanced associated domain) is central to the transcriptional activity required to drive proliferation and survival across several human cancers.^107^ Recently, using CRISPR/Cas9 loss-of-function screening, Edwards et al. revealed a role for YAP1/TAZ-TEAD activation in impairing the antiproliferative and proapoptotic effects of a KRAS^G12C^ inhibitor in several *KRAS*^*G12C*^-mutant cancer cell lines.^288^ In this work, YAP1 was found to overcome KRAS dependency through two TEAD-dependent mechanisms: first, via the transcriptional stimulation of ERK-independent genes, and second, through the activation of the PI3K-AKT-mTOR signaling pathway. As a rescue experiment, the inhibition of TEAD synergistically enhanced the antitumor activity of the KRASG12C inhibitor in vitro and in vivo. Similarly, a recent study evaluated the role of GNE-7883, a new pan-TEAD inhibitor that limits chromatin accessibility at TEAD motifs.^289^ GNE-7883 was evaluated in diverse preclinical models and was shown to overcome both intrinsic and acquired resistance to KRAS^G12C^ inhibitors, thus setting the stage for future clinical applications.

##### Immunotherapy-based combinational strategies

In addition to its oncogenic effects, *KRAS* mutations affect the TME composition^207,290,291^: KRAS activation has been shown to increase the production of neutrophil chemoattractants (CXCL1, CXCL2, and CXCL5), the recruitment of M1 macrophages, the attraction and induction of immunosuppressive regulatory T cells, and the enhancement of tumor infiltration of myeloid-derived stem cells (MDSCs). In preclinical in vivo PDAC mouse models, mutant *KRAS* was shown to promote tumor immune evasion, whereas loss of KRAS activity blocked tumor growth in immunocompetent mice.^292^ Accordingly, KRAS inhibition is thought to also affect the TME, and such observations have laid the groundwork for the combination of KRAS inhibition with either ICIs or other TME-modifying agents.

### SHP2 inhibition revisited

As discussed earlier, SHP2/KRAS coinhibition alters cancer cell signaling and modulates adaptive resistance to KRAS inhibition. SHP2, in addition to affecting cell signaling, has also been shown to induce favorable effects within the TME, such as depletion of MDSCs and enhancement of cytotoxic T cells (via the IL-6, JAK-STAT3, TNF, and IFNγ signaling pathways).^207^ These changes are typically associated with increased sensitivity to PD-1 blockade. In a preclinical study using PDAC and NSCLC cell lines, SHP2 inhibition and KRASG12C inhibition were synergistic through direct antiangiogenic and antisignaling actions as well as through the modulation of immune TME components.^207,293^ Subsequent work in genetically engineered mice revealed that SHP2 inhibition in KRAS-mutated NSCLC depleted M2-line macrophages and increased T-cell and B-cell infiltration.^294^ These observations suggest that oncogenic KRAS promotes immune escape via immunosuppressive modulation of the TME and that the addition of SHP2 inhibition can mitigate this escape mechanism.^87,295,296^ The translation of these preclinical findings into the clinical arena is awaited.

### Combining KRAS and immune checkpoint inhibition

Currently, ICIs are approved for several indications across various cancers.^297^ In advanced NSCLC, ICIs are usually the standard of care (alone or in combination with chemotherapy) in patients who lack driver mutations.^298^ This population includes patients with KRAS mutations, for whom sotorasib and adagrasib are approved only as second-line agents and who are likely to receive ICIs. Considering the immunomodulatory impact of KRAS on the TME, these patients may benefit less from ICIs, and combining KRAS inhibitors with ICIs appears to be an attractive rationale. Similarly, in non-NSCLC patients for whom KRAS inhibition is indicated, the inhibitor may reinvigorate the TME, making the addition of ICIs a sound approach to explore.

In *KRAS*^*G12C*^-mutant mouse models, the combination of sotorasib with ICIs resulted in synergistic tumor cell killing, along with proinflammatory changes reported within the TME.^87^ Mice that experienced a complete response (surrogate of “cure”) were rechallenged with KRASG12C cells and rejected them, highlighting the mounting of an adequate memory immune cell response against common antigens.^87^ Similarly, the combination of adagrasib and an anti-PD-1 antibody resulted in a durable antitumor response in a subcutaneous syngeneic *KRAS*^*G12C*^-mutant mouse model.^299^ Currently, this approach of combining a KRAS^G12C^ inhibitor with an ICI is being evaluated in several clinical trials across *KRAS*^*G12C*^-mutant solid tumors (Table 2), including with sotorasib in CodeBreaK 100 (NCT03600883) and CodeBreaK 101 (NCT04185883), adagrasib in KRYSTAL-1 (NCT03785249) and KRYSTAL-7 (NCT04613596), GDC-6036 in GO42144 (NCT04449874) and JDQ443 in KontRASt-01 (NCT04699188). While the combination of sotorasib and ICI has been limited by significant hepatotoxicity, this does not appear to be the case with adagrasib or second-generation inhibitors. Notably, the efficacy and safety data of olomorasib (a second-generation KRASG12C inhibitor) in combination with pembrolizumab were recently presented at the World Conference on Lung Cancer 2024 conference.^300^ In this phase 1 trial, olomorasib was evaluated in combination with pembrolizumab and standard-of-care chemotherapy in treatment-naïve, *KRAS*^*G12C*^-mutant NSCLC patients with any level of PD-L1 expression. Preliminary results revealed an 85% DCR and 50% ORR and a manageable safety profile consistent with what is reported in the current standard of care. A global registration study investigating this combination is currently ongoing (SUNRAY-01; NCT06119581). Other strategies involving *KRAS* mRNA modalities in combination with ICIs have also been reported.^233^

Other triple combinations are being evaluated on the basis of the newly acquired understanding of the impact of KRAS on the TME. For example, molecules that disrupt the CXCR1/2-MDSC axis may be combined with ICIs and KRAS/or SHP2 inhibitors to reverse MDSC-mediated immunosuppression.^301,302^ Other combinations of SHP2, KRAS, and IC inhibitors may also reshape the TME and suppress the adaptive signaling of cancer cells, which diminishes sensitivity to KRAS^G12C^ inhibition.^207,303,304^

In addition to the abovementioned combinations, novel approaches have been described to target immune suppression mechanisms and KRAS-driven immune evasion. For example, KRAS inhibition has been shown to increase the infiltration of regulatory T cells (T-regs) that suppress antitumor immunity.^87,295,305^ Thus, current strategies involve the selective depletion of these tumor-infiltrating T-regs through the targeting of specific cell surface markers (CD25 or CCR8).^306^ Direct targeting of KRAS-driven immune evasion mechanisms has also been explored. For example, KRAS drives the expression of COX-2, resulting in the production of the immune-suppressive prostaglandin E2, and the inhibition of COX-2 or prostaglandin E2 has been shown to increase sensitivity to anti-PD-1 when combined with KRAS inhibitors.^307,308^ Different therapeutic strategies exploiting the impact of KRAS inhibition on tumor immunity have been recently reviewed.^309^

### RAS-on-state inhibitors

Unlike other codon 12 mutations, the impact of G12C mutations on intrinsic GTPase hydrolytic activity is limited but still induces insensitivity to GAP, hence leading KRAS^G12C^ to cycle predominantly into its active GTP-bound form. As discussed earlier, both adagrasib and sotorasib covalently bind the mutant cysteine residue of the KRAS^G12C^-GDP form, blocking it in its inactive state and hence preventing its cycling to the active GTP-bound state. However, preclinical work has shown that inhibition of KRAS^G12C^ results in feedback reactivation of upstream RTKs, which can result in the activation of wild-type RAS isoforms, thus leading to increased RAS-GTP levels and decreasing the efficacy of KRAS^G12C^ inhibitors.^310^ Similarly, KRAS^G12D^ has been shown to have even lower intrinsic GTPase activity than does KRAS^G12C^, hence resulting in most KRAS^G12D^ proteins being in the active GTP-bound form. Preclinical work involving KRASG12D-mutant PDAC cell lines treated with MRTX1133, a KRAS^G12D^ selective inhibitor, revealed that pERK suppression, which was observed at 2 h, rebounded by 48 h after treatment, highlighting the same adaptive mechanism observed earlier with *KRAS*^*G12C*^ inhibitors.^310,311^ These observations have led to drug development efforts toward the design of agents that can address adaptive RAS signaling mechanisms that rely on increased active-state, wild-type, and mutant RAS proteins.

Such agents that bind and inhibit KRAS in its GDP (“off”) and GTP (“on”) bound states are referred to as RAS-on inhibitors. MRTX1133 is perhaps the first described molecule that targets both GDP and GTP-bound KRAS^G12D^ via noncovalent binding to the switch 2 pocket, resulting in successful inhibition of KRAS-mediated signaling and substantial tumor regression in xenograft mouse models.^311^ Further work using NMR spectroscopy and competitive bioluminescence resonance energy transfer (BRET)-based engagement assays demonstrated that the switch 2 pocket of several other non-G12C *KRAS* mutants can also be targeted, independent of the GDP-bound form.^312^ More recent strategies have also been described to target *KRAS* mutants in both their active and inactive forms via a mechanism shared by the immunosuppressant cyclosporin^313^: cyclosporin binds to the chaperone protein cyclophilin A (peptidyl-prolyl cis-trans isomerase A), and the nascent complex, in turn, binds to and inhibits the serine/threonine phosphatase calcineurin.^314^ Accordingly, several compounds that bind KRAS in both its GTP and GDP states, as well as cyclophilin, leading to an inhibitory trimeric (or “tri-complex”), have been explored.^193,315–318^ Importantly, since cyclophilin A is expressed ubiquitously, diverse RAS-mutant tumor types can be virtually amenable to this tri-complex strategy.^319^

Examples of KRAS-on inhibitors include RM-018, a KRAS^G12C^ covalent inhibitor that interacts with cyclophilin A and targets KRAS^G12C^ in its GTP-bound state.^193^ In a published clinical case report, a patient with KRAS^G12C^ NSCLC who developed resistance to adagrasib was treated with RM-018, which could overcome resistance.^193^ Resistance in this case was mediated via the recycling of KRAS into its GTP-active form, leading to reactivation of the RAS-MAPK signaling pathway. Similar tri-complex KRAS^G12C^-on inhibitors, including RMC-6291, are superior to adagrasib.^316^ The same concept and principles behind this targeting approach were applied to other KRAS mutations, leading to translational application in early-phase clinical trials: RMC-9805, for example, targets KRAS^G12D^ in its GTP-bound state, with recent data supporting its safe and effective use in KRAS^G12D^-mutant PDAC patients. In this phase 1/1b trial (NCT06040541), the ORR was 30%, and the DCR was 80%, with no reported dose-limiting toxicities, and the maximum tolerated dose was not reached.^320^ RMC-8839 is a first-in-class KRAS-on inhibitor that targets KRAS^G13C^ in its GTP-bound state, highlighting again the increased dependence of KRAS^G13C^-mutant cancers on wild-type RAS isoforms.^321^ Both of these compounds were recently presented and discussed at the EORTC-NCI-AACR symposium on Molecular Targets and Cancer Therapeutics in October 2024.

These promising strategies against multiple KRAS mutants have led to further exploitation of the RAS-targeting approach, with efforts geared toward developing pan-KRAS-on inhibitors. The use of structure-guided design and optimization approaches led to the development of RMC-7977, the first orally bioavailable, multiselective, pan-KRAS-mutant GTP-bound inhibitor.^315^ Preclinically, RMC-7977 has demonstrated potent activity against RAS-addicted tumors with various *RAS* genotypes, particularly against *KRAS*^*G12*^ mutations: treatment with RMC-7977 led to tumor regression across diverse preclinical models, including those resistant to KRASG12C inhibitors.^315^ This RAS-ON, a multiselective inhibitor, could target multiple oncogenic and wild-type RAS isoforms. RMC-6236 is a related RAS-ON, multiselective inhibitor that has recently entered clinical evaluation in patients with *KRAS*-mutant PDAC (NCT05379985): in the second-line setting, metastatic PDAC patients treated with RMC-6236 had a median PFS of 8.1 months (in KRASG12X; 95% CI: 5.9-not evaluable) and 7.6 months (all-RAS-mutated disease), and in those with third-line or later-stage PDAC, the median PFS was 4.2 months.^322^ While this was not a comparative study, the benchmark median PFS for second-line standard-of-care therapy was approximately 2-3.5 months, whereas it was 1.9 months for third-line therapy or beyond. ORRs were 20% and 27% at 14+ and 20+ weeks for KRASG12X patients and 21% and 26%, respectively, for those with any RAS mutation. This is compared with a 9% estimated benchmark ORR with standard-of-care therapy. The DCRs were 87% and 88% for the KRASG12X and all-RAS mutations, respectively, at 14+ weeks. In terms of dosing, RMC-6236 was administered at doses ranging from 160 mg to 300 mg, with a 300 mg dose, once daily as part of a 21-day treatment cycle, resulting in the highest steady-state AUC in almost all patients. In terms of toxicity, any-grade treatment-related adverse effects (TRAEs) occurred in 96% of patients, with 22% experiencing grade 3 or higher effects. The most common TRAEs that occurred in at least 10% of patients were rash (any grade, 87%; grade ≥3, 6%), diarrhea (46%; 2%), nausea (43%; 0%), stomatitis or mucositis (38%; 2%), vomiting (28%; 0%), fatigue (17%; 1%), and paronychia (10%; 0%). These resulted in dose interruptions and reductions for 27% and 11% of patients, respectively, with none requiring discontinuation. While the median OS was not evaluable in either subset of patients, these findings are promising for a disease with a grim prognosis and provide ground evidence for the initiation of RASolute-302, the first global, randomized phase III trial of RMC-6236 in second-line metastatic PDAC patients. Notably, prior data from patients with KRASG12X-mutated NSCLC (n = 40) revealed a relatively high ORR (38%) and DCR (34%), with a short median time to response (1.4 months vs. 3.3 months for patients with PDAC).^323^

**Fig. 5:**
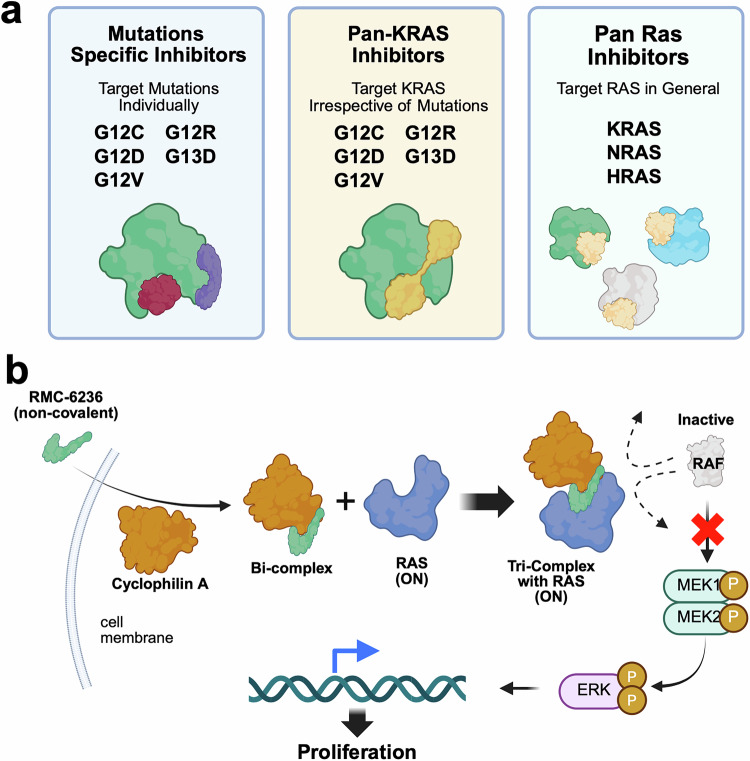
Mechanism of KRAS-On-State Targeting. KRAS-on-state inhibitors are classified into mutation-specific inhibitors (target mutations individually; blue panel), pan-KRAS inhibitors (targeting KRAS irrespective of mutations; beige panel), and pan-RAS inhibitors (targeting KRAS, NRAS, and HRAS; light green panel) (**a**). On-state targeting includes the binding of the inhibitor to cyclophilin to form a bicomplex, which in turn binds to KRAS in its active-GTP form, resulting in the inhibition of the Tri-complex KRAS (**b**). Created in BioRender.com

Two novel, highly potent, and orally bioavailable tri-complex pan-RAS-mutli(ON) inhibitors have been described.^324^ In vitro investigations of RCZY-690 and RCZY-680 revealed high binding affinities for cyclophilin A, with potent nanomolar EC50 values for binding to the G12C, G12V, and G12D KRAS mutants in a homogeneous time-resolved fluorescence (HTRF) assay. In a pancreatic KRAS G12D-mutant PDAC xenograft model, both compounds showed potent antitumor efficacy, with sustained inhibition of intratumoral pERK.^324^ Figure 5 highlights the mechanism of RAS(ON) targeting.

### KRAS mRNA vaccines and other nucleic acid-based approaches

Nucleic acid-based therapies are emerging approaches to address KRAS-mutant tumors, with therapies potentially tailored on the basis of gene sequence information, hence bypassing previously intractable targets with conventional small molecules. These modalities include several approaches: small interfering RNA (siRNA), antisense oligo (ASO)-based approaches, CRISPR‒Cas-based therapies, and mRNA-based vaccines. These approaches selectively modulate gene expression at the transcriptomic level, mediate precise genome editing, and can deliver transcripts that encode tumor-associated antigens, hence harnessing the power of the immune system.^325–327^

### siRNA-based approach

siRNAs are short double-stranded RNAs that directly and complementarily bind to target mRNAs, leading to their degradation via the RNA-induced silencing complex (RISC).^328^ This approach has been specifically exploited both in vitro and in vivo to target mutant *KRAS*, resulting in a selective reduction in the growth of KRAS-dependent tumors.^329–333^ However, translation into the clinical arena has been limited by challenges related to siRNA stability (and vulnerability to nucleases in the circulatory system), poor pharmacokinetics, and issues related to drug delivery.^334^ Research has thus focused on delivery formulations using lipid nanoparticles and exosomes with two small interfering RNA (siRNA)-based KRAS drugs, which are currently undergoing clinical trials: siG12D KRAS-LODER uses a miniature biodegradable polymeric matrix for controlled drug delivery of the siRNA, and iExosomes containing siG12D siRNA use nanosized extracellular vesicles within a lipid bilayer membrane.^335^ More recently, preclinical work using pananti-KRAS siRNA modalities embedded with functionalized lipid nanoparticles has been the subject of research, with liver accumulation being a major limitation.^333,336^ Other si-KRAS delivery systems are also being explored, including peptide-modified bioresponsive chimeric polymersomes, aerosol inhalation nanoparticles, and antibody-pan-KRAS siRNA conjugates (AOCs).^337–339^

### Antisense oligo (ASO)-based approach

Like siRNAs, ASOs are single-stranded oligonucleotides that complementarily bind to specific RNA sequences, inducing RNA cleavage, as well as steric blockage of the translational machinery. Currently, ASO-based KRAS targeting has been limited to preclinical work, with no ASO therapies tested in clinical trials. Like the challenge faced by siRNA drugs, drug delivery is a major hindering factor in the clinical development of ASO-based, KRAS-targeting therapies. One exception is AZD4785, a 16-nucleotide antisense oligo that binds complementarily to the 3′ untranslated region of KRAS mRNA.^340^ This was the only agent tested in a clinical setting, as part of a phase 1 trial of 28 patients with advanced solid tumors, with no published results, and no further clinical trials were conducted or published.

### CRISPR‒Cas-based approach

The CRISPR‒Cas system is a widely used genome-editing technology that relies on designed guide RNA (gRNA) to specifically recognize and target selected genes.^341,342^ Several in vitro studies using CRISPR-based approaches to target and disrupt KRAS have been conducted in KRAS-mutant cancer.^343–347^ Off-target mutations, toxicity, and delivery efficacy have been the main challenges limiting the use of CRISPR-based modalities, as unintentional editing of a genome carries profound, long-term complications.^348^ More recently, CRISPR-Cas9 base editors have been developed to enable precise base conversion, without DNA cleavage, to correct specific KRAS mutations.^349^ Cas13 is another Cas protein that specifically recognizes and cleaves RNA instead of DNA, making it a potent alternative approach to control the expression of oncogenic transcripts in cancer cells.^346,350,351^ In vitro and in vivo studies revealed that the use of the CRISPR-Cas13a system could specifically downregulate KRAS^G12D^ mRNA without affecting WT mRNA, resulting in significant antitumor activity.^346^

### mRNA vaccines and immunomodulating oligonucleotides

mRNA vaccines operate on the basis that an individual’s tumors carry a unique set of somatic mutations that can be recognized by the host immune system as “nonself”, thus triggering a tumor-targeted immune response. An mRNA-based vaccine trains the immune system to recognize and initiate humoral and T-cell-mediated immunity against cancer-specific antigens.^352^ While this concept of a “personalized vaccine” was initially explored in patients with melanoma,^353,354^ it offers a platform to generate epitope-directed vaccines, including KRAS epitopes.

mRNA-5671 was developed in 2018 as an mRNA vaccine against the most prevalent KRAS mutations (G12C, G12D, and G12V): the mRNA-expressing KRAS epitope, delivered through an encapsulated lipid nanoparticle, was evaluated in a phase 1 clinical trial (NCT03948763) as an intramuscular injection administered every 3 weeks for a total of 9 cycles. The trial evaluated mRNA-5671 alone in combination with pembrolizumab in 70 patients with KRAS-mutant advanced/metastatic NSCLC, CRC, and PDAC. While the study has been completed, the results from this trial have not yet been published.

Compared with traditional, linear mRNA vaccines, circular RNAs (circRNAs) form covalently closed structures produced by the back-splicing of precursor mRNAs, hence exhibiting increased stability, away from exonuclease-mediated degradation.^355^ Although engineered circRNAs have not yet been studied for their ability to specifically target KRAS-mutant tumors, promising antitumor effects against solid tumors via the expression of an immunogen that induces the host immune response have been demonstrated.^356^

mRNA vaccines thus provide a new opportunity for the treatment of KRAS-mutant cancer as a promising therapeutic strategy to trigger robust, tumor-specific immune responses.

### Immunomodulating oligonucleotides

Despite the promise of the ongoing development of mRNA vaccines, the oncogenic KRAS-induced reprogramming of the TME that facilitates cancer cell immune evasion remains a challenge.^357^ To that end, adjuvant therapies aimed at increasing the immunogenicity of a cancer vaccine, including Toll-like receptor (TLR) agonists, have been explored.^358,359^ One example is ELI-002, a novel immunomodulating oligonucleotide comprising a lymph node-targeted amphiphile (Amph)-modified synthetic oligonucleotide (CpG-7909) that acts as a TLR9 agonist in combination with a mixture of Amph-conjugated peptide-based antigens that target mutant KRAS.^360^ Upon binding to TLR9, CpG-7909 stimulates B-cell proliferation, increases the production of antigen-specific antibodies, and induces interferon-alpha production and interleukin-10 secretion.^361^ ELI-002 was evaluated in a phase 1 trial as a subcutaneous injection and was administered to 22 patients with KRAS/NRAS-mutant PDAC in the adjuvant setting for minimal residual disease (NCT05726864). The trial demonstrated safety (with no dose-limiting toxicities and mostly grade 1 adverse events), and phase 1-2 trials are currently ongoing to assess the efficacy of the treatment in KRAS/NRAS-mutant strains (NCT04853017).

*In summary, nucleic acid-based strategies, including mRNA vaccines, have shown promise as potential alternatives to conventional therapies targeting KRAS-mutant tumors. Progress regarding drug stability and delivery has resulted in improved clinical applicability. Moreover, these agents offer promising options for addressing acquired resistance in KRAS-driven tumors. Current ongoing trials involving nucleic acid-based therapy targeting KRAS tumors are summarized in Table* 2.

### KRAS adoptive T-cell therapy

CD8+ T cells eliminate cancer cells because of their ability to recognize and target nonself-peptides produced by hotspot mutations in the *KRAS* and *TP53* oncogenes known as “neoantigens”.^362,363^ These peptides, deemed foreign/nonself are presented to TCRs by MHC-I molecules.^362^ Adoptive cell therapy (ACT) is the process of isolating these CD8+ tumor-infiltrating lymphocytes (TILs), expanding them ex vivo, and then reintroducing them to the patient. While initially used with melanoma,^364,365^ ACT strategies have more recently been extended to several cancer types, including *KRAS*-mutant tumors. Clinical trials exploring the use of ACT for epithelial cancers are primarily neoantigen-reactive TCR gene-engineered T-cell therapies using reactive host/autologous TCRs or allogenic TCRs for individual and shared hotspot mutations, respectively. These TCRs are introduced via a vector into the patient’s peripheral blood-derived T cells.^366^

This was based on initial work by Tran et al., who reported the regression of metastatic lesions in the lungs of a 50-year-old female with widespread CRC following polyclonal infusion of HLA-C*08:02-restricted CD8+ T cells with reactivity against 4 neopeptides of KRAS G12D. After surgical resection of 3 metastatic lung lesions, the other lung nodules were cultured to isolate tumor-infiltrating lymphocytes (TILs), which were then tested for reactivity against neoantigens. CD8+ T cells within cultured TILs were reactive to KRAS^G12D^ mutants. These cells were subsequently expanded and infused after a prior nonmyeloablative lymphodepletion regimen in the patient. Nine months later, a lesion progressed due to immune evasion mediated by the loss of the chromosome 6 haplotype encoding HLA-C*08:02 MHC 1, which is required for recognition by KRAS G12D-reactive T cells.^367^ Regression of widespread metastatic foci in a patient with advanced pancreatic cancer was also noted with treatment using HLA-C*08:02-restricted autologous T cells.^368^

The identification of HLA-C*08:02 restrictions for targeting KRAS G12D neoepitopes heralds an opportunity for potential therapy for common driver mutations in cancers.^366^ KRAS G12 mutations are commonly found in PDAC, CRC, and NSCLC. With HLA-C*08:02 being detected in 8% of Caucasians and 11% of African Americans in the United States, thousands of patients can be eligible for T-cell therapy targeting KRAS G12D cancers.^363,369^ A similar restriction involving HLA-A*11:01 has been reported for KRAS G12V and KRAS G12D in mouse models, although these have not yet been tested in clinical trials.^234,370^ The HLA-A*11:01 allele is found in 14% of U.S. Caucasians, 23% of African Americans, and 50% of Han Chinese.^369^ TCR gene therapy using allogenic TCRs to target KRAS or TP53 is readily available, as the receptors are off-the-shelf; however, specific mutations and HLA combinations must be present.^366^ Using a sensitive in vitro stimulation and enrichment technique, researchers have isolated CD4+ and CD8+ memory T cells specific to shared/somatic oncogenic mutations, such as KRAS G12D and KRAS G12V, as well as unique mutations from the peripheral blood of patients with epithelial cell cancers.^371^ This signals the potential for eliminating TIL harvesting in ACT.^369^ To overcome the poor immunogenicity and paucity of TILs in PDAC and the loss of specificity toward mutant *KRAS* with the use of K562-based artificial antigen-presenting cells in PDAC, researchers introduced TMG-T cells (tandem mini gene-modified T cells). These are modified autologous T cells that express tandem mini genes, offering a reliable way to expand rare neoantigen-specific T cells and increasing the potential for effective ACT in PDAC.^372^ A more diverse library of TCRs that can react to a wider variety of mutations and HLA restrictions will enable off-the-shelf therapies rather than relying solely on personalized treatments. The specific targeting of mutated KRAS makes T-cell therapy particularly appealing, although further research is needed.

Other MHC-I-restricted mutant *KRAS* epitopes have since been characterized, including TCRs that recognize the G12V-HLA-A*03:01, G12V-HLA-A*11:01, and G12R-HLA-B*07:02 complexes, which can be redirected toward KRAS-mutant cell lines of various histologies.^373^ In xenograft models of metastatic KRASG12V-mutant NSCLC, ACT of TCR-engineered CD8+ T cells specific for G12V-HLA-A*03:01 or G12V-HLA-A*11:01 showed efficacy.^373^ Building on this knowledge, a very recently published work highlighted the successful development of a chimeric antigen receptor T-cell (CAR-T) construct using engineered binders that target the oncogenic KRAS G12V mutations presented by peptide‒MHC complexes, which are then incorporated into CAR-T cells (mKRAS neoCARs).^374^ This preclinical work revealed efficacy in xenograft models of metastatic lung, pancreatic, and renal cell cancer. Interestingly, this study further demonstrated the safety and efficacy of activating these CAR-T cells in vivo through inducible secretion of IL-12 and T-cell receptor deletion.

Data on effective KRAS adoptive T-cell therapy remains limited to case reports and preclinical work, as highlighted above. While theoretically promising, several challenges face the field, including HLA restriction, whereby TCRs must be matched to a patient’s HLA type, tumor heterogeneity, immune evasion, limited persistence of T cells, off-tumor effects (cytokine release syndrome), and complexity of manufacturing and patient-specific customization.^375^ Challenges common in *KRAS* mutation-based vaccine design and adoptive T-cell therapy should also be addressed, namely, those related to the immunogenicity of KRAS-mutated tumors; for example, Skoulidis et al. highlighted the heterogeneous and distinct immune profiles of KRAS-mutant NSCLC, which are primarily dictated by the co-occurrence of specific genomic alterations, ultimately defining *KRAS* immunogenicity and therapeutic vulnerabilities.^376^ In addition to the immunosuppressive impact of certain co-occurring genomic alterations on the TME, other significant limitations and challenges to KRAS immune targeting include the reduction or absence of MHC-I expression.^377^ For example, genetic changes in β-2-microglobulin (B2M), including point mutations, loss of heterozygosity, or expression downregulation, have been described.^378–380^ B2M is an essential binding partner of the MHC-I heavy chain needed for antigen presentation, and alterations in its expression result in MHC-I instability and the subsequent reduction/absence of antigen-presenting ability, immunogenicity, and immunotherapy effectiveness. Similarly, additional factors have been shown to downregulate MHC-based antigen presentation via histone modifications. For example, histone H3 lysine 27 trimethylation (H3K27me3) has been shown to induce the downregulation of genes involved in antigen presentation, and the loss of WHSC1, a histone methyltransferase responsible for the demethylation of lysine 36 on histone H3 (H3K36me2), also impacts MHC-I expression.^381^ Despite an encouraging theoretical framework and preclinical data, several challenges remain to be addressed as far as the immunogenicity of KRAS mutations is concerned.

### KRAS PROTACs and molecular glues

There are two classes of protein degraders, namely, proteolysis-targeting chimeras (PROTACs) and molecular glue degraders (MGDs).^382^ PROTACs are heterobifunctional compounds that are composed of two main components connected by a linker: (i) a target binder, also known as the warhead, which is a ligand that binds proteins of interest (POIs), and (ii) an ER binder, which is a ligand for E3 ubiquitin ligases such as cereblon (CRBN), Von Hippel–Lindau (VHL), and the cellular inhibitor of apoptosis protein (cIAP).^382–384^ The ATP-driven ubiquitin proteasome system (UPS) is responsible for protein degradation, the removal of misfolded or aged housekeeping proteins, the cellular immune response by processing antigenic peptides, and cell cycle regulation through cyclin degradation in cells.^383,385^ PROTACs are designed to overtake endogenous E3-mediated protein degradation within the cell, bringing the POI and E3 ligase in close spatial proximity and creating artificial accessibility to promote polyubiquitination.^386^ E2 ligases are then recruited by E3 ligases, which mediate the transfer of ubiquitin from E2 to the target protein. Afterwards, the PROTAC complex dissociates, and the polyubiquitinated protein is designated for complete degradation by the 26S proteasome.^383^ PROTAC activities are event-driven and iterative (recyclable), offering many advantages over traditional molecular inhibitors. These include lower dosage requirements and extended dosing intervals, catalytic effects, increased potency and duration of action, increased selectivity minimizing toxicity, effectiveness against drug resistance mechanisms, the ability to target nonenzymatic functions, and an expanded range of target possibilities.^386,387^ The high molecular weight and large polar surface area of peptide PROTACs are responsible for their low cell permeability and solubility and low potential stability, limiting their pharmacokinetics.^382,386,388^ All-small-molecule PROTACs were subsequently developed that penetrate cells and cause intracellular protein degradation of the androgen receptor bound to the selective androgen modulator receptor (SARM) and the murine double minute 2 (MDM2) ligand called nutlin, which is connected via a PEG-based linker.^389^ Other small-molecule PROTACs include MDM2,^390^ VHL,^391^ CRBN^392^ and c LAP-1-based PROTACs.^393^

The first PROTAC targeting endogenous KRASG12C was LC-2, which recruits the MRTX849 warhead and a VHL-based E3 ligase to covalently bind to G12C-mutated KRAS, causing its degradation via suppression of the MAPK pathway. This effect was observed in both the homozygous and heterozygous KRASG12C cell lines.^394^ This interaction, however, was irreversible and covalent, abolishing the iterative nature central to PROTACs and preventing recycling of the degraders. The first reversible covalent PROTAC YF-135 was reported with an MRTX845 POI ligand, and a VHL E3 ligase ligand was reported.^395^ Other high-affinity nonimmunologic molecules, such as designed ankyrin repeat proteins (DARPins) and intracellular single variable domains (iDABs), have been used to develop PROTACs when small-molecule PROTACs cannot be used.^396^ DARPins and iDABs have been fused with the VHL E3 ligase and CHIP E3 ligase, respectively. The KRAS-specific DARPin degrader induces cell death in both the mutant and wild-type KRAS strains by binding to the allosteric lobe of KRAS, whereas the pan-RAS degrader iDAB affects proliferation irrespective of the RAS mutation, i.e., N/H/KRAS.^397^ A study on peptide PROTACs demonstrated that speckle-type POZ protein (SPOP) is the most effective E3 ligase for RAS-specific degradation. Researchers then created constructs of SPOP fused with RAS biologics such as NS1 Mb, K27, K55 DARPins, R11.1.6 Sso7d, and c-RAF RBD-CRD. With the exception of K55, all the other combinations resulted in the depletion of KRAS expression. The K27 PROTAC had the strongest effect on KRAS depletion in PDAC (KRAS G12D) cells, which degraded KRAS in the following order: KRASWT > KRASG12C > KRASG12D > KRASG12V. Furthermore, K27 SPOP has increased proclivity toward GDP-bound/inactive KRAS, as treatment with sotorasib resulted in increased degradation of KRAS G12C.^398^ A monobody, known as 12VC1, binds to the active forms of KRAS G12V and G12C, with 400 times greater affinity than WT KRAS. Compared with 12VC1 alone, the fusion of 12VC1 with VHL degrades and suppresses mutant KRAS better. Compared with DARPin-based PROTACs, the VHL-12VC1 fusion has a slower rate of degradation.^399^

Immunomodulatory drugs such as thalidomide and its derivatives, such as lenalidomide and pomalidomide, are among the most recognized synthetic molecular glue degraders (MGDs) approved for the treatment of multiple myeloma, AML, and other hematological diseases.^314,382^ They bind the cereblon protein (CRBN), which, along with Cullin4A (cul4) and damage-specific DNA-binding protein 1 (DDB1), forms the Cullin RING Ligase 4 (CRL4) complex, which is responsible for protein degradation. The binding of small molecules such as thalidomide reprograms the substrate specificity of CRL4, allowing it to target new substrates for degradation. MGDs are small molecules that stabilize protein‒protein interactions between E3s and new protein targets.^382,388^ New classes of MGDs have been discovered, such as E7820, Indisulam, and chloroquinoxaline sulfonamides, which bind the DDB1 and CUL4-associated factor 15 (DCAF15) subunits of CRL4, resulting in the degradation of the RNA-binding motif protein 39 (RBM39) splicing factor.^400^ CDK inhibitors (CR8 and HQ461) bind to the DDB1 subunit of CRL4, causing cyclin K degradation via its binding to CDK12, subverting the need for substrate recognition.^401,402^

ACB13 is the first kind of selective, potent, and in vivo pan-KRAS degrader capable of targeting a wide range of highly prevalent KRAS-mutant cancers. It was developed by enhancing the stability and durability of the VHL-PROTAC-KRAS complex through a structure-based design approach. PROTAC-mediated KRAS degradation has 10-fold greater potency than traditional KRAS inhibitors do and results in long-term suppression of MAPK signaling. ACB13 continues to suppress mutant KRAS levels even after the drug has been cleared from the body.^403,404^ Pan-KRAS degraders, if successfully translated into clinical practice, can be valuable tools in the arsenal of oncologists for overcoming treatment-induced resistance to KRAS inhibitors and treating KRAS-driven malignancies.^404,405^

## Summary: Ongoing clinical research strategies targeting KRAS

Throughout this comprehensive review, several treatment strategies and approaches to inhibit KRAS have been described. While some inhibitors have already been approved for clinical use, many ongoing trials are evaluating potential strategies to target KRAS. Key strategies include direct targeting of KRAS^G12C^, which has been challenged by adaptive resistance, targeting of other mutations (KRASG12D), the development of RAS(ON) inhibitors, and the use of combinational strategies. More recently, nucleic acid-based therapies (including siRNAs, mRNA vaccines, CRISPR‒Cas systems), immunotherapy approaches, and PROTACs have entered early-phase clinical trials.

**Fig. 6:**
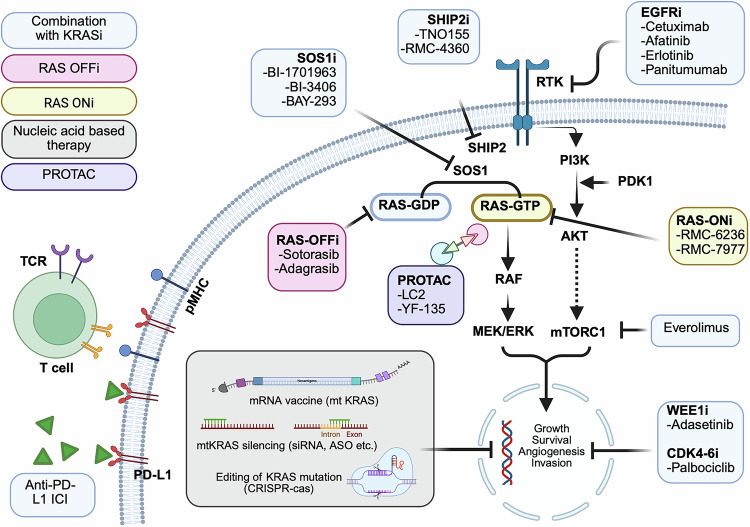
Summary of KRAS-targeting strategies. This figure summarizes different KRAS-targeting strategies beyond the currently approved small-molecule inhibitors (pink). Strategies include combination approaches with KRAS inhibitors (light blue), RAS-ON inhibition (yellow), the use of PRTOAC (purple), and nucleic acid-based therapies (gray), such as mRNA vaccines, siRNAs, and CRISPR‒Cas-based therapies. TCR T-cell receptor, pMHC peptide-major histocompatibility complex. Created in BioRender.com

While the clinical trial landscape is constantly evolving, Table 2 below summarizes the most up-to-date landmark and currently ongoing preclinical and clinical trials aimed at targeting KRAS mutations. Figure 6 summarizes the different therapeutic strategies, in addition to small-molecule inhibitors.

## Conclusions and future directions

Over the past few years, we have witnessed exponential growth in the arsenal of inhibitors, novel targeting methods and strategies, and new combination approaches aimed at first, bypassing the inevitable adaptive resistance to currently approved KRAS inhibitors, and second, leveraging the impact of inhibiting KRAS, using increasing knowledge regarding its partners, and intricate relationships with the TME. Despite these advances, only two KRAS^G12C^ inhibitors are currently approved for clinical use and remain far from curative. In this work, we present a comprehensive summary of the challenges facing KRAS inhibition, along with several promising ongoing preclinical and clinical studies evaluating novel strategies. Our work also highlights the complexity of RAS targeting, the “holy grail” of many malignancies, while providing a framework for the diverse approaches being evaluated. Our work describes strategies for overcoming resistance mechanisms through coinhibition of several nodes in the RAS pathway, as well as combination strategies to coinhibit parallel pathways. While such coinhibitory approaches have shown promising preclinical and early clinical efficacy, issues pertaining to added toxicity need to be closely monitored. Similarly, measuring efficacy in terms of objective response rates awaits a clinically meaningful translation in terms of survival benefit. Leveraging immunotherapeutic strategies via TME modulation and other mechanisms we described earlier is likely to induce durable antitumor immune responses and could bring us closer to curative drugs. Thus, a careful combination of direct KRAS-targeting agents and immune checkpoint inhibitors is warranted.

This wealth of novel KRAS inhibition strategies in preclinical and clinical development calls for a focus on and prioritization of key research priorities. Moving forward, and given the existing approval of two direct KRAS inhibitors that one can build on, the most significant challenge facing KRAS inhibition is overcoming resistance that limits the depth and duration of response. From our discussion of the literature, it has become clear that KRAS inhibitor monotherapy does not confer long-term effectiveness and necessitates novel combination therapies. Challenges in combination strategies include heterogeneity of these mechanisms and divergence among patients and tumors. Hence, future research should prioritize understanding these mechanisms and developing personalized approaches based on individual tumor molecular signatures for optimal combination selection, as well as novel technology to monitor response and detect resistance early on. Circulating tumor DNA (ctDNA) may provide a platform for this type of investigation. Clinically, personalized selection of therapeutic combinations may be carried out via a master protocol design that evaluates multiple combination strategies in parallel and via a basket‒trial approach on the basis of the patient’s molecular profile. In the world of overcoming resistance and personalizing therapies, several questions remain unanswered: which drug design is best in overcoming primary resistance? Are all the mechanisms of adaptive resistance equal, and accordingly, which mechanism should first be targeted to prevent resistance and improve the efficacy of KRAS inhibition? Can drivers of primary and secondary resistance be identified at diagnosis and subsequently used as potential biomarkers to predict response, anticipate the emergence of resistance, and thus tailor therapy?

In addition to exploring strategies to overcome resistance to direct KRAS inhibitors, the development of novel approaches is also warranted. Efforts are ongoing in the arena of immune targeting of KRAS mutations through the development of therapeutic vaccines and adoptive T-cell therapies (TCR-T and CAR-T cells). These advances are made possible through novel approaches: TCR-T (TCR-engineered T-cell therapy), for example, involves engineering a patient’s own T cells to express a TCR that recognizes a specific KRAS-mutant peptide presented by the patient’s HLA molecule.^406–408^ On the other hand, efforts are also ongoing toward the universal design of TCR protein drugs through the building of TCR libraries with broad patient coverage or the engineering of TCR mimic antibodies (TCRm) that bind to the same MHC-peptide complexes as TCRs while providing an alternative way to target KRAS.^409–411^ Other promising strategies involve the specific targeting of KRAS peptide-MHC complexes through the design of de novo, highly specific binders for these complexes, and such strategies have been used for the development of the KRAS CAR-T cells described earlier.^374,412,413^

While nucleic acid-based therapies, immunotherapies, and PROTACs are exciting and potentially promising, they remain in the early phases of development. Efforts should subsequently focus on other direct inhibitors with mechanistically distinct approaches than the two currently approved, mutant-selective “off” allosteric inhibitors. The RAS “on” inhibitors, in that context, are thus worthy of attention from the KRAS community, given the unanticipated clinical efficacy of the multi-RAS inhibitor RMC-6236. This class of inhibitors not only provides pathways for primary resistance but also, through their pan-KRAS multisectivity, allows the targeting of other non-G12C *KRAS* mutations, such as KRAS G12D, which is most prevalent in lethal diseases such as PDAC. However, whether the resistance mechanisms associated with KRAS^G12C^ inhibitors also drive resistance to KRAS “on” inhibitors remains unknown. Similarly, questions about novel resistance mechanisms are also likely to arise.

In conclusion, KRAS targeting has evolved significantly, from the era of being an “undruggable” target to the current state where several compounds are being evaluated in early-stage trials. The novel approaches we described herein are promising for achieving the long-standing goal of curing *KRAS*-mutant malignancies. At this time, RAS(ON) inhibitors appear to be a promising strategy, perhaps leading to a race in the clinical trial arena.
